# Ulcerative Colitis-Derived Colonoid Culture: A Multi-Mineral-Approach to Improve Barrier Protein Expression

**DOI:** 10.3389/fcell.2020.577221

**Published:** 2020-11-23

**Authors:** Muhammad N. Aslam, Shannon D. McClintock, Durga Attili, Shailja Pandya, Humza Rehman, Daniyal M. Nadeem, Mohamed Ali H. Jawad-Makki, Areeba H. Rizvi, Maliha M. Berner, Michael K. Dame, Danielle Kim Turgeon, James Varani

**Affiliations:** ^1^Department of Pathology, The University of Michigan Medical School, Ann Arbor, MI, United States; ^2^Department of Internal Medicine (The Division of Gastroenterology), The University of Michigan Medical School, Ann Arbor, MI, United States

**Keywords:** basement membrane, calcium, cell barrier, colonoid, desmosome, organoid culture, proteomics, ulcerative colitis

## Abstract

**Background:**

Recent studies demonstrated that Aquamin^®^, a calcium-, magnesium-rich, multi-mineral natural product, improves barrier structure and function in colonoids obtained from the tissue of healthy subjects. The goal of the present study was to determine if the colonic barrier could be improved in tissue from subjects with ulcerative colitis (UC).

**Methods:**

Colonoid cultures were established with colon biopsies from 9 individuals with UC. The colonoids were then incubated for a 2-week period under control conditions (in culture medium with a final calcium concentration of 0.25 mM) or in the same medium supplemented with Aquamin^®^ to provide 1.5 – 4.5 mM calcium. Effects on differentiation and barrier protein expression were determined using several approaches: phase-contrast and scanning electron microscopy, quantitative histology and immunohistology, mass spectrometry-based proteome assessment and transmission electron microscopy.

**Results:**

Although there were no gross changes in colonoid appearance, there was an increase in lumen diameter and wall thickness on histology and greater expression of cytokeratin 20 (CK20) along with reduced expression of Ki67 by quantitative immunohistology observed with intervention. In parallel, upregulation of several differentiation-related proteins was seen in a proteomic screen with the intervention. Aquamin^®^-treated colonoids demonstrated a modest up-regulation of tight junctional proteins but stronger induction of adherens junction and desmosomal proteins. Increased desmosomes were seen at the ultrastructural level. Proteomic analysis demonstrated increased expression of several basement membrane proteins and hemidesmosomal components. Proteins expressed at the apical surface (mucins and trefoils) were also increased as were several additional proteins with anti-microbial activity or that modulate inflammation. Finally, several transporter proteins that affect electrolyte balance (and, thereby affect water resorption) were increased. At the same time, growth and cell cycle regulatory proteins (Ki67, nucleophosmin, and stathmin) were significantly down-regulated. Laminin interactions, matrix formation and extracellular matrix organization were the top three up-regulated pathways with the intervention.

**Conclusion:**

A majority of individuals including patients with UC do not reach the recommended daily intake for calcium and other minerals. To the extent that such deficiencies might contribute to the weakening of the colonic barrier, the findings employing UC tissue-derived colonoids here suggest that adequate mineral intake might improve the colonic barrier.

## Introduction

An intact colonic barrier is necessary for gastrointestinal health (Coskun, 2014; France and Turner, 2017). Colonic barrier dysfunction is a consistent feature of inflammatory bowel disease, seen in both Crohn’s disease and ulcerative colitis (UC) (Salim Sa and Söderholm, 2011; Antoni et al., 2014; Vivinus-Nebot et al., 2014; Lee et al., 2018). Barrier dysfunction is also seen in irritable bowel syndrome and celiac disease, as well as in conjunction with acute bacterial infection of the gastrointestinal tract (Pearson et al., 1982; Dunlop et al., 2006; Moreira et al., 2012). While barrier dysfunction can result from toxic insults or from inflammatory attack on the epithelial cells lining the colon, it is now becoming recognized that pre-existing weaknesses in the gastrointestinal barrier may predispose the tissue to inflammation and injury. In case of long-term injury, this chronic inflammation can lead to colon cancer as seen in UC-associated cancer (Ekbom et al., 1990; Ullman and Itzkowitz, 2011). Therefore, enhancing the colonic barrier may have beneficial effects beyond mitigating chronic inflammation (Chelakkot et al., 2018). Histologic evidence of reduced colonic inflammation and lower polyp incidence was demonstrated in two long-term polyp-prevention studies by a multi-mineral dietary approach in mice (Aslam et al., 2010, 2012).

Environmental factors, especially diet, have a significant impact on colonic barrier health. A high-fat diet has been shown to increase intestinal permeability as has hyperglycemia (Moreira et al., 2012; Thaiss et al., 2018). Most studies have focused on tight junctional complexes as the primary regulator of permeability, and high-fat or high-sugar intake has been shown to affect these structures (Moreira et al., 2012; Thaiss et al., 2018). Similarly, it has been demonstrated that intestinal desmoglein-2, an adhesion molecule of desmosomes, is required for the maintenance of intestinal barrier and its deficiency cause increased barrier permeability (Schlegel et al., 2020). Our own work has demonstrated the importance of calcium as a regulator of the colonic barrier. In a recent study, we showed that increasing the extracellular calcium concentration from a basal level of 0.25 mM to as high as 3.0 mM in human colon organoid (colonoid) culture had a modest effect on expression of tight junction proteins, but dramatically up-regulated desmosomal proteins (desmoglein-2, desmocollin-2, and desmoplakin) (Attili et al., 2019). This was accompanied by an actual increase in desmosomes seen at the ultrastructural level. A substantial increase in multiple basement membrane components including laminin α, β, and γ chains as well as nidogen and perlecan (basement membrane heparin sulfate proteoglycan) was also observed in calcium-treated colonoids (Attili et al., 2019).

As part of our investigation, we carried out assays to assess the effects of calcium-supplementation on *trans*-epithelial electrical resistance (TEER) and colonoid cohesion (McClintock et al., 2020). Consistent with tight junctional protein expression under control conditions, TEER values were high to begin with. In spite of the high background, electrical resistance still increased in response to calcium-supplementation. Colonoid cohesion, consistent with the considerable increase in desmosomes and basement membrane proteins in response to intervention, was low in basal medium but increased substantially in treated colonoids (McClintock et al., 2020). Of interest, while calcium provided as a single agent was effective, a calcium-, magnesium-, and multiple trace element-rich natural product (Aquamin^®^) was more effective than calcium alone (McClintock et al., 2020).

These findings indicate that colonoid culture technology can provide a useful tool for elucidating factors that affect barrier structure and function in the colon. The present study extends this work. Here we describe results with colonoid cultures established with tissue from UC patients. The UC-derived colonoids were maintained under control conditions (0.25 mM calcium) or treated with Aquamin^®^ – the same multi-mineral natural product used in our previous studies - at amounts bringing the final calcium level to 1.5 – 4.5 mM. Effects on components of the barrier were determined using several approaches.

## Materials and Methods

### Aquamin^®^

Aquamin^®^ is a multi-mineral natural product obtained from the mineralized remains of red marine algae of the Lithothamnion family. Aquamin^®^-Soluble, the form used here, has been used in our previous colonoid studies (McClintock et al., 2018, 2020; Attili et al., 2019). It consists of calcium (approximately 12% by weight), a calcium to magnesium ratio of 12:1 and detectable levels of 72 other trace minerals – essentially all of the minerals accumulated in the algae fronds from seawater. During growth, the mineralized fronds break off from the viable algae and fall to the ocean floor from where they are harvested. After harvest, the fronds are processed to remove organic material, leaving only the mineral remains. Aquamin^®^ is sold as a food supplement (GRAS 00028) and is used in various products for human consumption in Europe, Asia, Australia, and North America [Marigot Ltd, Cork, IR]. A single batch of Aquamin^®^ was used in the present study. Mineral analysis of this batch was provided by an independent laboratory (Advanced Laboratories; Salt Lake City, UT, United States) using Inductively Coupled Plasma Optical Emission Spectrometry. Mineral composition of Aquamin^®^ is provided in Supplementary Table 1.

### Establishment of Colonoid Cultures From UC Biopsies

De-identified colonic tissue (2.5-mm biopsies) was obtained from the sigmoid colon of eleven subjects during surveillance colonoscopy. The study was approved by the Institutional Review Board (IRBMED) at the University of Michigan and all subjects provided written informed consent prior to endoscopy.

Colonoid cultures were established as described in several recent reports with tissue from healthy subjects (Dame et al., 2018; McClintock et al., 2018, 2020; Tsai et al., 2018; Attili et al., 2019). Briefly, biopsies were finely minced on ice using a #21 scalpel and seeded into Matrigel (Corning), prepared at 8 mg/mL. A size of 40 μm (diameter) was considered ideal for tissue fragment size. During the expansion phase (between 2 and 5 weeks), colonoids were incubated in L-WRN medium. This medium consists of 50% fresh Advanced DMEM – F12 medium (Gibco) as its base and 50% of the same medium that had been conditioned (with 20% Fetal Bovine Serum- FBS [Gibco], resulting in final concentration of 10% FBS) with the Wnt-, R-spondin- and Noggin-transfected L-cells as described by Miyoshi and Stappenbeck (Miyoshi and Stappenbeck, 2013). The calcium concentration is 1.0 mM. The medium was supplemented with human recombinant epidermal growth factor (EGF) at 100 ng/mL (R&D Systems), 2 mM GlutaMax (Invitrogen), 10 mM HEPES (Invitrogen), N2 media supplement (Invitrogen), B27 Supplement minus vitamin A (Invitrogen), 1 mM *N*-Acetyl-L-cysteine (Sigma), 100 μg/ml Primocin (InvivoGen), and the small molecule inhibitors: 500 nM A 83-01 (Tocris), a TGF-β inhibitor, 10 μM SB 202190 (Sigma), a p38 inhibitor and 10 μM Y27632 (Tocris) as a ROCK inhibitor. For the first 10 days of culture, the medium was also supplemented with 2.5 μM CHIR9902 (Tocris) to enhance Wnt signaling. During the initial isolation and expansion phase colonoids were, typically, expanded as follows: Passage (P)0 - culture initiation; P1 – 1:1 “clean-up” passage to remove debris and non-epithelial components; P2 – 1:2 expansion; P3 – 1:1 clean-up” passage to remove debris and any remaining non-epithelial components; P4 – 1:2 expansion; P5 – 1:2 or greater expansion depending upon colonoid density (cryopreservation occurred at this point).

Experiments were carried out with P6–P8 cultures. For the experimental phase, L-WRN culture medium was diluted 1:4 with KGM Gold, a serum-free culture medium (Lonza) designed for epithelial cell proliferation. The final serum concentration in the medium was 2.5% and final calcium concentration was 0.25 mM. This “control treatment medium” was compared to the same medium supplemented with Aquamin^®^ to provide a final calcium concentration of 1.5, 2.1, 3.0, or 4.5 mM. Fresh medium and treatments were provided at 2-day intervals. The last 2-day medium collected before harvest at day-14 were saved for cytokine assessment. At the end of the in-life phase, colonoid samples were evaluated for gross appearance using both phase-contrast microscopy and scanning electron microscopy (SEM). Other samples were prepared for histology and examined at the light-microscopic level after staining with hematoxylin and eosin. The same preparation was used for immunohistology. Additional samples were fixed in glutaraldehyde and used for transmission electron microscopy (TEM). Finally, samples were frozen in liquid nitrogen and allocated to proteomic analysis.

### Phase-Contrast Microscopy

Phase-contrast microscopy (Hoffman Modulation Contrast - Olympus IX70 with a DP71 digital camera) was used to assess colonoids in whole-mount for change in size and shape. Images were taken twice weekly and at the end of the in-life phase of the study.

### Histology, Immunohistology and Morphometric Analysis

At the end of the in-culture phase, colonoids were isolated from Matrigel using 2 mM EDTA and fixed in 10% formalin for 1 h. Fixed colonoids were suspended in HistoGel (Thermo Scientific) and then processed for histology (i.e., hematoxylin and eosin staining) or for immunohistology. For immunostaining, freshly cut sections (5–6 μ) were rehydrated and subjected to heat-induced epitope retrieval with high pH or low pH FLEX TRS Retrieval buffer (Agilent Technologies, 154 #K8004; Santa Clara, CA, United States) for 20 min. After peroxidase blocking, antibodies were applied at appropriate dilutions at room temperature for 30 or 60 min depending on the manufacturer’s recommendation. The FLEX HRP EnVision System (Agilent Technologies) was used for detection with a 10-min DAB chromagen application. Supplementary Table 2 provides a list of antibodies used, their source and additional experimental details.

The sections of immunostained colonoid tissue on glass slides were digitized using the Aperio AT2 whole slide scanner (Leica Biosystems) at a resolution of 0.5 μm per pixel with 20X objective. These scanned images were housed on a server and accessed using Leica Aperio eSlide Manager (Version 12.3.2.5030), a digital pathology management software. The digitized histological sections were viewed and analyzed using Aperio ImageScope (Version 12.4.0.5043 by Leica Biosystems), a slide viewing software. Brightfield Immunohistochemistry Image Analysis tools (Leica) were used to quantify immunostains used in this study. Aperio Nuclear Algorithm (v9) was used for proliferation marker (Ki67) quantification. This algorithm measures intensity of nuclear staining and separates those into very intense to no nuclear staining (3+, 2+, 1+, and 0 respectively). Nuclei with higher intensity (3+ and 2+) were used here for comparison. The Aperio Positive Pixel Count Algorithm (v9) was used to quantify expression of the other markers. It quantifies number and the intensity of pixels of a specific color in a digitized image. Positivity was calculated with respective numbers of strong positive and positive pixels against total pixels.

### SEM and TEM

Colonoid specimens were fixed *in situ* in 2.5 percent glutaraldehyde in 0.1 M Sorensen’s buffer, pH 7.4, overnight at 4°C. After subsequent processing for SEM or TEM as described previously (Bleavins et al., 2012), samples for SEM were then mounted on stubs, allowed to off-gas in a vacuum desiccator for at least 2 h and sputter coated with gold. Samples were examined with an Amray 1910 FE Scanning Electron Microscope and digitally imaged using Semicaps 2000 software. For TEM, ultra-thin sections were examined using a Philips CM100 electron microscope at 60 kV. Images were recorded digitally using a Hamamatsu ORCA-HR digital camera system operated with AMT software (Advanced Microscopy Techniques Corp., Danvers, MA, United States).

### Differential Proteomic Analysis

Colonoids were isolated from Matrigel using 2 mM EDTA for 15 min and then exposed to Radioimmunoprecipitation assay (RIPA) – lysis and extraction buffer (Pierce, # 89901; ThermoFisher Scientific) for protein isolation, as described in our previous reports (McClintock et al., 2018; Attili et al., 2019). Proteomic experiments were carried out in the Proteomics Resource Facility (PRF) in the Department of Pathology at the University of Michigan, employing mass spectrometry-based Tandem Mass Tag (TMT, ThermoFisher Scientific). For this, three subjects were assessed separately using TMT sixplex kit for each subject. Fifty micrograms of colonoid protein from each condition was digested separately with trypsin and individual samples labeled with one of 6 isobaric mass tags following the manufacturer’s protocol. After labeling, equal amounts of peptide from each condition were mixed together. In order to achieve in-depth characterization of the proteome, the labeled peptides were fractionated using 2D-LC (basic pH reverse-phase separation followed by acidic pH reverse phase) and analyzed on a high-resolution, tribrid mass spectrometer (Orbitrap Fusion Tribrid, ThermoFisher Scientific) using conditions optimized at the PRF. MultiNotch MS3 analysis was employed to obtain accurate quantitation of the identified proteins/peptides (McAlister et al., 2014). Data analysis was performed using Proteome Discoverer (v2.3, ThermoFisher Scientific). MS2 spectra were searched against UniProt human protein database (20353 sequences; downloaded on 06/20/2019) using the following search parameters: MS1 and MS2 tolerance were set to 10 ppm and 0.6 Da, respectively; carbamidomethylation of cysteines (57.02146 Da) and TMT labeling of lysine and N-termini of peptides (229.16293 Da) were considered static modifications; oxidation of methionine (15.9949 Da) and deamidation of asparagine and glutamine (0.98401 Da) were considered variable. Identified proteins and peptides were filtered to retain only those that passed ≤ 2% false discovery rate (FDR) threshold of detection. Quantitation was performed using high-quality MS3 spectra (Average signal-to-noise ratio of 9 and <40% isolation interference). Differential protein expression between conditions, normalizing to control (0.25 mM calcium) for each subject’s specimens separately was established using edgeR (Robinson et al., 2010). Then, resulting data from the three data sets were combined. Proteins names were retrieved using Uniprot.org, and Reactome v70 (reactome.org) was used for pathway enrichment analyses (Jassal et al., 2020). Only Proteins with a ≤2% FDR confidence of detection were included in the analyses. STRING database - v11 (string-db.org) was used for additional enrichment analyses and to detect protein-protein interactions. For the enrichment analysis, STRING implements well-known classification systems such as Gene Ontology (GO) and provide information related to molecular functions and biological processes. Our initial analysis was targeted toward differentiation, barrier-related, cell-cell and cell-matrix adhesion proteins. Follow-up analysis involved an unbiased proteome-wide screen of all proteins modified by intervention in relation to control. The mass spectrometry proteomics data deposited to the ProteomeXchange Consortium via the PRIDE partner repository with the dataset identifier PXD020244 (Perez-Riverol et al., 2019).

### Cytokine Assay

Approximately 400 μL of culture medium was collected at the time of harvest. A panel of pro-inflammatory cytokines, including tumor necrosis factor-α (TNF-α), interleukin-1β (IL-1β), IL-6, IL-8 and macrophage chemotactic peptide-1 (MCP-1) was assessed. Levels of each were determined using the Bio-Plex bead-based cytokine assay from Bio-Rad Laboratories (Hercules, CA, United States).

### Ethics Statement

For the current study, de-identified colon tissue was collected during surveillance colonoscopy. Tissue collection was done under a protocol reviewed and approved by the Institutional Review Board at the University of Michigan (IRBMED). Patients’ protected health information was not shared with the investigators. Each subject signed a written informed consent document prior to the procedure allowing the use of the colonic tissue for research purposes.

### Statistical Analyses

Group means and standard deviations were obtained for discrete morphological and immunohistochemical features as well as for individual protein values (proteomic analysis) and cytokine values. Data generated in this way were analyzed by one-way analysis of variance (ANOVA) followed by Dunnett’s multiple comparisons test for comparison using GraphPad Prism (v 8.3). Supplementary Table 3 provides a complete list of *p*-values calculated to determine statistical significance for quantitative histology and immunohistology. Proteome Discoverer also computed an abundance ratio *p*-value based on a nested design of biological replicates. It presented the p value of the sample group calculated by running the Tukey HSD test (*post hoc*) after an ANOVA test. Pathways enrichment data reflect Reactome-generated *p*-values based on the number of entities identified in a given pathway as compared to total proteins responsible for that pathway. A binomial test was applied within Reactome to calculate the probability shown for each result, and the *p*-values were corrected for the multiple testing (Benjamini–Hochberg procedure) that arises from evaluating the submitted list of identifiers against every pathway. For STRING enrichment analysis high (<1 percent) FDR stringency was used. For the enrichment analysis, the whole genome statistical background was assumed. Data were considered significant at *p* < 0.05.

## Results

### Establishment of Colonoid Cultures From UC Biopsies

Colonic tissue (duplicate 2.5-mm biopsies) was obtained by endoscopy from the sigmoid colon of 11 subjects with a diagnosis of UC. Five of the subjects had active disease at time of biopsy. The other six participants were considered to be in remission; though all had disease-related symptoms (or active flare) within the past 4 years of tissue collection. Tissue was obtained from the site of inflammation in subjects with active disease. Relevant demographic and clinical information is presented in Table 1. Tissue was established in colonoid culture as described in the section “Materials and Methods.” Two of the active-lesion samples failed to grow and were eventually discarded. Successful cultures were established from the remaining nine tissue samples. The “in-remission” colonic tissue samples were ready for experimentation after 2 or 3 weeks of culture – similar to what was typically seen with tissue from healthy individuals (Attili et al., 2019). Tissue from “active lesion” samples required 3–5 weeks to reach this stage.

**TABLE 1:** Demographic information, disease status, pathology report, and culture notes.

ID	Age	Gender	Year of diagnosis	Last flare	Pathology report	Culture notes
UC 124	61	M	2015	2016	Quiescent chronic colitis	Expanded in 2 weeks
UC 125	28	F	1999	2017	Quiescent chronic colitis	Expanded in 2 weeks
UC 127	56	F	2012	2014	No histological abnormality	Expanded in 2 weeks
UC 128	39	F	2014	Current Flare	Active ulcerative colitis	Expanded in 2 weeks
UC 129	60	M	2008	Current Flare	Active ulcerative colitis	Expanded in 5 weeks
UC 130	52	F	2010	2016	Quiescent chronic colitis	Expanded in 4 weeks
UC 131	50	M	2003	2016	Quiescent chronic colitis	Expanded in 3 weeks
UC 132	58	M	2015	Current Flare	Severe active ulcerative colitis	Did not grow
UC 133	24	F	2015	Current Flare	Severe active ulcerative colitis	Did not grow
UC 134	61	M	2018	2018	No histological abnormality	Expanded in 3 weeks
UC 135	75	M	2005	2016	Active ulcerative colitis	Expanded in 3 weeks

*Biopsies were taken from the sigmoid colon during surveillance colonoscopies. All patients were on maintenance therapy at the time of biopsy. Last flare refers to clinical presentation with active disease. Pathological assessment of colon biopsies was done at the time of collection.*

### Effects of Mineral Intervention on Gross and Microscopic Features

**FIGURE 1:**
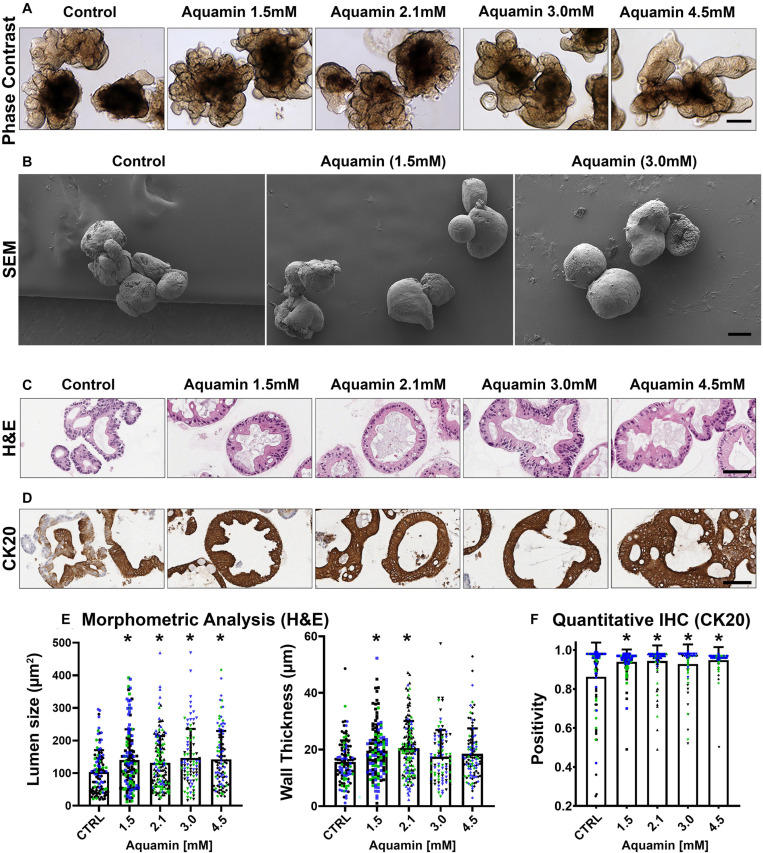
UC colonoid appearance. Phase-contrast microscopy **(A)**: At the end of the incubation period, intact colonoids were examined by phase-contrast microscopy. Colonoids were present as thick-walled structures with few surface buds. A wide range of sizes and shapes were seen under all conditions. Bar = 200 μm. Scanning electron microscopy **(B)**: Scanning electron microscopy confirmed the presence of smooth surface and few buds in colonoids maintained under low-calcium conditions (Control). Aquamin^®^-treated colonoids were similar to those maintained in the low-calcium medium. Bar = 100 μm. Histological features **(C)**: At the end of the incubation period, colonoids were examined by light microscopy after staining with hematoxylin and eosin. Under low-calcium conditions (Control), the colonoids were found to be crypts of varying size with a single layer of epithelial cells surrounding a central lumen. Tiny crypts (with as few as 20 cells in cross section) were seen. In the presence of Aquamin^®^, larger crypts made up of columnar epithelial cells surrounding a large, often irregular-shaped lumen were seen. Goblet cells were apparent. Bar = 100 μm. CK20 expression **(D)**: Immunohistology revealed high-expression of CK20 under all conditions. Bar = 100 μm. Quantification of morphological features and CK20 expression **(E,F)**: Lumen size and wall thickness **(E)**: Means and standard deviations are based on pooled crypts analysis (87–143 individual crypts per condition) of 3 subjects. Asterisks (*) indicate statistical significance from control at *p* < 0.05. CK20 expression **(F)**: Means and standard deviations are based on *n* = 3 subjects and 81–120 individual crypts per condition. Asterisks (*) indicate statistical significance from control at *p* < 0.05. (Subject IDs: Black dots: Subject#1; Green dots: Subject#2; Blue dots: Subject#3).

Once established, colonoids were incubated for a 2-week period (with subculture at the end of week-one) under control conditions (0.25 mM calcium) or in the presence of the multi-mineral intervention formulated to provide 1.5 – 4.5 mM calcium. Phase-contrast images and SEM images taken at harvest (Figures 1A,B) identified no gross morphological changes attributable to the intervention. In both control and treated cultures, individual colonoids appeared as round or egg-shaped structures. Most colonoids consisted of a single structure but some were multi-lobed. In most places, the colonoids still had a layer of the Matrigel substrate attached, but in other places, the substrate was gone and individual colonoid cells could be seen. At the beginning of the treatment phase, and immediately after the first subculture, colonoid structures were approximately 40 μm in size (on average). Over the culture period, they increased to approximately 400 μm in diameter. Overall, UC-derived colonoid appearance and growth characteristics were similar to what has been reported previously with colonoids established from tissue of healthy individuals (Attili et al., 2019).

Although there were no apparent alterations in gross morphology with the intervention, a modest increase in differentiation features was revealed by quantitative histology and immunohistology. As seen in hematoxylin and eosin-stained tissue sections (Figure 1C), colonoids maintained for two weeks in the presence of the multi-mineral intervention had a larger lumen diameter and greater wall thickness than control. This was confirmed morphometrically (Figure 1E). Expression of the differentiation marker, cytokeratin 20 (CK20) (Figure 1D), was substantial under control conditions, but still increased (albeit, modestly) in response to treatment (Figure 1F). Supplementary Figure 1 provides low and high power phase-contrast and histological/immunohistological images of UC colonoids.

**FIGURE 2:**
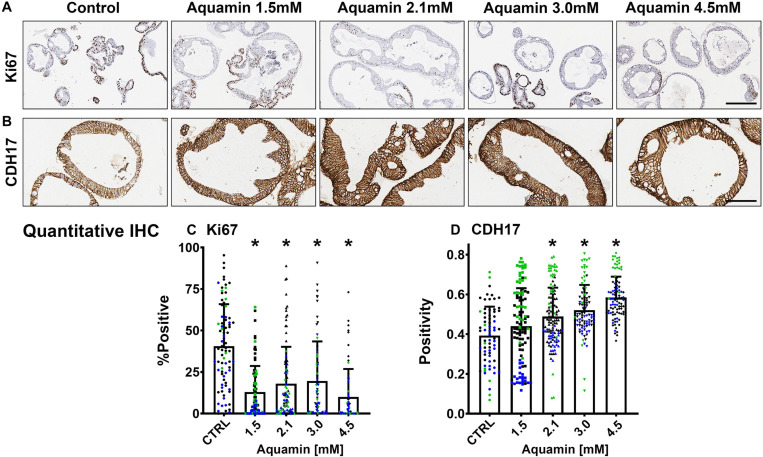
Ki67 and cadherin-17 expression by immunohistology. At the end of the incubation period, colonoids were examined after immunostaining of histological sections. Ki67 **(A)**; Bar = 200 μm and Cadherin-17 **(B)**; Bar = 100 μm. Quantitative assessment of Ki67 staining **(C)** is based on nuclear algorithm (v9) and pooled data represent means and standard deviations from *n* = 3 subjects and 36–78 individual crypts per condition. Asterisks (*) indicate statistical significance from control at *p* < 0.05. Cadherin-17 **(D)** values represent positivity (measured using Positive Pixel Value v9). Means and standard deviations are based on *n* = 3 subjects and 68–124 individual crypts per condition. Asterisks (*) indicate statistical significance from control at *p* < 0.05. CDH17: Cadherin-17. (Subject IDs: Black dots: Subject#1; Green dots: Subject#2; Blue dots: Subject#3).

Epithelial differentiation is linked with growth reduction (Anghileri and Tuffet-Anghileri, 1982). As part of our evaluation, we assessed Ki67 expression in control and treated colonoids. A decrease in Ki67 expression was seen with treatment (Figures 2A,C). Levels under both control and treated conditions were comparable to what was seen previously in colonoids from healthy subjects (Attili et al., 2019), but lower than what we observed with colon adenomas in colonoid culture (McClintock et al., 2018).

### Effects of Aquamin^®^ on Protein Expression Profile in UC-Derived Colonoids: Barrier Proteins

A Tandem Mass Tag mass spectrometry-based approach was used to assess the effects of Aquamin^®^ on proteins involved in differentiation and barrier formation (Garcia et al., 2018). Table 2 provides a list of proteins of interest. There are several things to note. First, CK20 up-regulation was seen with Aquamin^®^-treatment; this is consistent with the immunohistochemical expression data shown as part of Figure 1. A number of other differentiation-related proteins were also up-regulated in Aquamin^®^-treated colonoids (Table 2A), but only with CK80 was the level of up-regulation comparable to that of CK20. Several additional keratins (and other differentiation-related proteins) were also detected in the UC colonoids, but did not change significantly with treatment.

**TABLE 2A:** Proteins involved in differentiation and barrier formation.

		Aquamin			
Proteins	Genes	1.5 mM	2.1 mM	3.0 mM	4.5 mM
**Keratins and Differentiation-related**					
Epiplakin	EPPK1	1.18 ± 0.17	*1.32 ± 0.13	*1.32 ± 0.12	1.35 ± 0.26
Keratin, type I cytoskeletal 19	KRT19	*1.16 ± 0.01	*1.44 ± 0.16	1.26 ± 0.19	*1.25 ± 0.13
Keratin, type I cytoskeletal 20	KRT20	*1.48 ± 0.24	*1.66 ± 0.15	*1.78 ± 0.23	*1.97 ± 0.50
Keratin, type II cytoskeletal 8	KRT8	*1.25 ± 0.10	*1.52 ± 0.09	*1.43 ± 0.04	*1.49 ± 0.10
Keratin, type II cytoskeletal 80	KRT80	*1.36 ± 0.10	1.44 ± 1.09	1.96 ± 0.92	1.69 ± 0.51
**Tight junction**					
Claudin-3	CLDN3	1.08 ± 0.04	*1.16 ± 0.04	1.05 ± 0.05	1.00 ± 0.09
Claudin-4	CLDN4	1.22 ± 0.16	*1.36 ± 0.08	*1.36 ± 0.10	*1.40 ± 0.18
Claudin-7	CLDN7	*1.26 ± 0.14	1.17 ± 0.30	1.26 ± 0.25	1.26 ± 0.21
Occludin	OCLN	0.97 ± 0.02	0.92 ± 0.03	0.90 ± 0.02	0.85 ± 0.02
Tight junction protein ZO-1	TJP1	0.96 ± 0.08	0.99 ± 0.09	0.95 ± 0.09	0.95 ± 0.10
Tight junction protein ZO-2	TJP2	0.89 ± 0.08	0.84 ± 0.12	0.83 ± 0.13	0.81 ± 0.15
Tight junction protein ZO-3	TJP3	0.95 ± 0.07	0.92 ± 0.11	0.89 ± 0.11	0.86 ± 0.14
Myosin-14	MYH14	1.27 ± 0.27	1.35 ± 0.38	1.55 ± 0.47	*1.53 ± 0.33
**Adherens junction**					
Cadherin-17	CDH17	*2.83 ± 0.22	*2.95 ± 0.60	*3.24 ± 0.67	*3.62 ± 0.70
Protocadherin-1	PCDH1	1.53 ± 0.43	1.75 ± 0.63	1.75 ± 0.81	2.02 ± 1.10
Cadherin-related family member 5	CDHR5	*1.51 ± 0.14	*1.61 ± 0.15	*1.77 ± 0.23	*1.95 ± 0.48
Cadherin-related family member 2	CDHR2	*1.71 ± 0.39	*1.75 ± 0.24	*2.00 ± 0.54	*2.09 ± 0.62
Cadherin-1 (*E*-cadherin)	CDH1	0.97 ± 0.07	0.93 ± 0.16	0.97 ± 0.18	0.96 ± 0.17
**Desmosomal and hemidesmosomal**					
Desmoglein-2	DSG2	*2.07 ± 0.17	*2.10 ± 0.25	*2.28 ± 0.37	*2.36 ± 0.40
Desmoplakin	DSP	*1.36 ± 0.20	*1.64 ± 0.12	*1.69 ± 0.08	*1.81 ± 0.09
Desmocollin-2	DSC2	*1.39 ± 0.07	*1.35 ± 0.11	*1.39 ± 0.13	*1.43 ± 0.12
Junction plakoglobin	JUP	*1.60 ± 0.18	*1.71 ± 0.12	*1.77 ± 0.13	*1.77 ± 0.11
Plectin	PLEC	*1.27 ± 0.10	*1.45 ± 0.08	*1.43 ± 0.14	*1.51 ± 0.03
Dystonin	DST	1.03 ± 0.12	1.11 ± 0.11	1.15 ± 0.14	1.19 ± 0.16
**Basement membrane, ECM molecules and CEACAMs**					
Laminin subunit alpha-1	LAMA1	*2.00 ± 0.62	*2.15 ± 0.38	*2.00 ± 0.49	*2.59 ± 0.44
Laminin subunit beta-1	LAMB1	*2.14 ± 0.70	*2.20 ± 0.48	*2.12 ± 0.56	*2.85 ± 0.42
Laminin subunit gamma-1	LAMC1	*2.03 ± 0.58	*2.07 ± 0.37	*2.01 ± 0.47	*2.68 ± 0.30
Laminin subunit beta-2	LAMB2	*1.91 ± 0.49	*2.03 ± 0.34	*1.90 ± 0.30	*2.32 ± 0.30
Laminin subunit beta-3	LAMB3	1.08 ± 0.05	*1.23 ± 0.08	*1.23 ± 0.12	1.25 ± 0.16
Nidogen-1 (Entactin)	NID1	1.70 ± 0.49	*1.66 ± 0.37	*1.72 ± 0.37	*2.11 ± 0.28
Perlecan	HSPG2	*1.13 ± 0.01	*1.23 ± 0.04	*1.24 ± 0.05	*1.32 ± 0.10
Integrin alpha-V	ITGAV	*1.18 ± 0.05	*1.21 ± 0.05	*1.29 ± 0.11	*1.34 ± 0.21
Integrin alpha-5	ITGA5	*1.69 ± 0.42	*1.80 ± 0.26	*2.21 ± 0.58	*2.27 ± 0.67
CEACAM 1	CEACAM1	1.72 ± 0.52	5.25 ± 4.20	2.01 ± 1.12	1.80 ± 1.18
CEACAM 5	CEACAM5	1.37 ± 0.29	*2.37 ± 0.51	1.81 ± 0.79	1.71 ± 0.82
CEACAM 6	CEACAM6	1.25 ± 0.35	*2.13 ± 0.35	1.50 ± 0.78	1.33 ± 0.80
CEACAM 7	CEACAM7	*1.84 ± 0.32	*2.00 ± 0.08	*2.23 ± 0.37	*2.44 ± 0.50
Extracellular matrix protein 1	ECM1	1.10 ± 0.26	1.03 ± 0.20	1.32 ± 0.20	*1.35 ± 0.09
CD44	CD44	*0.61 ± 0.01	*0.58 ± 0.12	*0.48 ± 0.12	*0.40 ± 0.09
**Mucins and Trefoils**					
Mucin-2	MUC2	1.07 ± 0.06	1.24 ± 0.24	1.09 ± 0.21	1.26 ± 0.35
Mucin-5B	MUC5B	*1.50 ± 0.08	*1.45 ± 0.18	*1.66 ± 0.30	1.62 ± 0.46
Mucin-12	MUC12	1.09 ± 0.14	1.06 ± 0.23	1.10 ± 0.20	1.17 ± 0.23
Mucin-13	MUC13	*1.37 ± 0.04	*1.36 ± 0.16	*1.53 ± 0.16	*1.72 ± 0.43
Trefoil factor 1	TFF1	1.18 ± 0.18	1.14 ± 0.15	1.10 ± 0.28	*1.19 ± 0.08
Trefoil factor 2	TFF2	*1.90 ± 0.26	1.55 ± 0.36	*1.82 ± 0.46	*1.92 ± 0.58
Trefoil factor 3	TFF3	1.22 ± 0.27	1.38 ± 0.29	1.14 ± 0.11	1.56 ± 0.37

Second, several proteins that make up tight junctions were detected but overall showed little change in response to mineral-intervention. Claudin-4 was the most responsive tight junction protein but was only increased by 1.4-fold at maximum. In contrast, desmosomal proteins were strongly up-regulated with increasing concentrations of the multi-mineral product. Several cadherin family members (adherens junction proteins) were also up-regulated. The major exception was cadherin-1 (*E*-cadherin), a protein closely associated with tight junction organization and that is known to have low sensitivity to changes in calcium concentration (Baumgartner, 2013).

**FIGURE 3:**
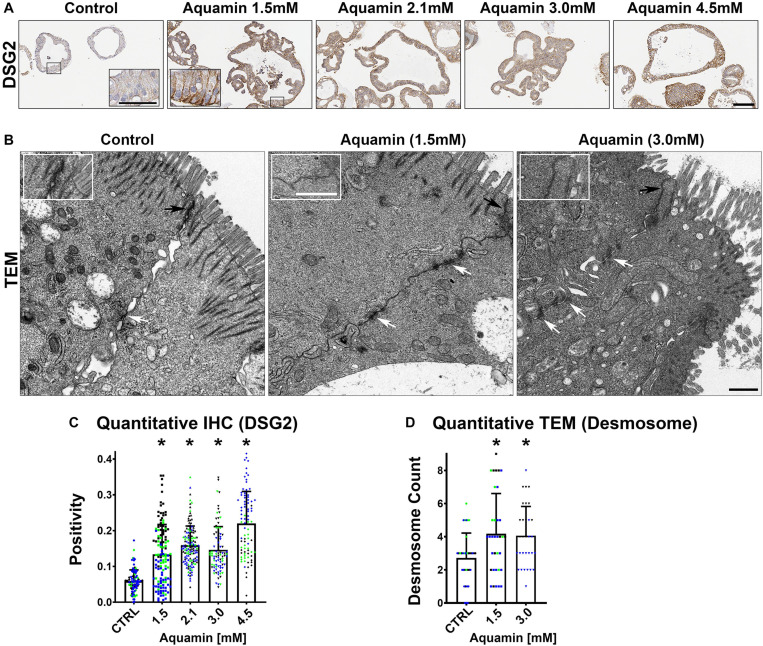
Desmoglein-2 and desmosomes. Immunohistology **(A)**: At the end of the incubation period, tissue sections were stained for desmoglein-2 and examined. Staining was diffuse and intracellular in colonoids maintained under low-calcium conditions. Staining was more intense in sections from Aquamin^®^-treated colonoids. Staining was prominent along the basolateral border in treated colonoids as seen in the inset. Bar = 200 μm, inset bar = 50 μm. Transmission electron microscopy **(B)**: At the end of the incubation period, ultra-thin sections were examined for desmosomes and other cell surface structures. Desmosomes were present in all conditions (white arrows) but a higher density of desmosomes along the lateral surface (cellular junctions between two cells) could be seen with intervention. Under all conditions, tight junctions were evident on the luminal surface (black arrows and insets). Magnification: 5,000X; Bars = 600 nm. Quantification of desmoglein-2 expression **(C)** and desmosome counts **(D)**: Immunostaining **(C)** results are means and standard deviations based on pooled crypt data from 3 subjects and 85–139 individual crypts per condition. Asterisks (*) indicate statistical significance from control at *p* < 0.05. Quantitative TEM **(D)**. The desmosome count was conducted at 5000X (*n* = 3 subjects with 7–18 ultra-structural images per subject) to obtain the actual number (means and SD) of desmosomes present in each high-power section. Asterisks indicate statistical significance from control at *p* < 0.05 level. (Subject IDs: Black dots: Subject#1; Green dots: Subject#2; Blue dots: Subject#3)

Immunohistology was used to confirm desmoglein-2 expression and change with intervention. As expected based on proteomic analysis, there was little expression under control conditions but strong up-regulation with intervention (Figures 3A,C). Control and Aquamin^®^-treated colonoid samples were also evaluated by TEM (Figure 3B). Desmosomes were visible under all conditions, but desmosome numbers increased measurably in treated colonoids as compared to control (white arrows) (Figures 3B,D). Desmosomes from the treated samples also tended to be wider and more electron-dense. As part of the immunohistological evaluation, cadherin-17 expression was evaluated as an adherens junction protein. Cadherin-17 was readily detected under low-calcium conditions in colonoid sections, but even with the high background, a significant increase with intervention was seen (Figures 2B,D).

In addition to proteins that constitute the major cell-cell adhesion complexes in the colonic epithelium, several additional proteins that contribute to cellular adhesive functions and to the structural barrier were also found to be up-regulated in the mineral-treated colonoids (Table 2). These include proteins making up the basement membrane (laminin α, β and γ chains, nidogen-1 [entactin] and perlecan [basement membrane-specific heparin sulfate proteoglycan]) as well as a protein that contributes to hemidesmosome formation and basement membrane attachment (e.g., plectin).

Proteins found at the apical surface – mucins and trefoils – are also shown in Table 2. Of these, certain proteins (e.g., mucin-5B and -13 as well as trefoil-1 and -2) were significantly elevated in response to treatment. Trefoils are proteins that interact with mucins to stabilize the mucous layer (Taupin and Podolsky, 2003; Aamann et al., 2014). Finally, a number of carcinoembryonic antigen-related cell adhesion molecules (CEACAMs) were strongly up-regulated in response to Aquamin^®^. This family of proteins demonstrated more subject-to-subject variability than was seen with most of the other moieties. In addition, whereas most of the other barrier proteins increased with increasing mineral concentration, several CEACAMs showed a peak response to Aquamin^®^ at a level providing 2.1 calcium and expression decreased at 3.0 and 4.5 mM except CEACAM7. Supplementary Figure 2 shows protein-protein interactions annotated by STRING database of cell-cell and cell-matrix adhesion proteins listed in Table 2. Different components of the cell barrier form distinct clusters characterized by strong interactions among these proteins.

### Effects of Aquamin^®^ on Proteins With Anti-microbial Activity and Proteins Related to Inflammation

In addition to the structural proteins described above, proteomic analysis also identified up-regulation of several proteins with known anti-microbial properties and modulation of additional proteins involved in the inflammatory response. Proteins with pro-inflammatory effects were decreased while proteins with anti-inflammatory, anti-oxidant and anti-apoptotic activity were uniformly increased (Table 2B). Among the list of up-regulated proteins were meprin A, a metalloproteinase whose presence in colon tissue is reduced in UC (Coskun et al., 2012), and two members of the solute carrier family (SLC5A1 and SLC15A1). These solute carrier proteins on the epithelial cell (intestinal) brush border serves as cotransporter and involved in uptake of glucose and peptides and is thought to have a protective role in UC (Yu et al., 2005; Wuensch et al., 2014). Finally, the major vitamin D binding protein was also strongly increased with intervention. Vitamin D signaling is important to calcium uptake and utilization and vitamin D-binding protein is also linked with decreased intestinal inflammation (Eloranta et al., 2011). Supplementary File 1 provide a list of citations to rationalize the inclusion of the anti-microbial and inflammation-modulating proteins in Table 2.

**TABLE 2B:** Inflammation-related proteins.

		Aquamin			
Proteins	Genes	1.5 mM	2.1 mM	3.0 mM	4.5 mM
**Anti-microbial peptides**					
Ly6/PLAUR domain-containing protein 8	LYPD8	*2.12 ± 0.22	*2.14 ± 0.50	*2.46 ± 0.40	*2.64 ± 0.93
NRAM2	SLC11A2	*1.91 ± 0.42	*2.20 ± 0.43	*2.56 ± 0.36	*2.68 ± 0.54
Lactotransferrin	LTF	2.46 ± 1.14	1.73 ± 0.24	*1.90 ± 0.02	2.20 ± 0.41
ATP-dependent RNA helicase DDX60	DDX60	1.47 ± 0.31	*1.57 ± 0.22	*1.73 ± 0.22	*1.75 ± 0.28
Cystatin-C	CST3	*1.36 ± 0.13	*1.36 ± 0.07	*1.44 ± 0.23	1.45 ± 0.31
ELMO domain-containing protein 2	ELMOD2	1.28 ± 0.26	*1.30 ± 0.11	1.36 ± 0.27	*1.43 ± 0.22
**Inflammation-related, anti-oxidant and anti-apoptotic**					
Tumor necrosis factor alpha-induced protein 2	TNFAIP2	1.04 ± 0.35	0.69 ± 0.51	0.65 ± 0.46	0.52 ± 0.42
Tumor necrosis factor alpha-induced protein 8	TNFAIP8	0.82 ± 0.25	1.00 ± 0.24	*0.82 ± 0.04	*0.74 ± 0.07
Apoptosis-associated speck-like protein	PYCARD	*0.79 ± 0.07	*0.71 ± 0.08	*0.63 ± 0.06	*0.59 ± 0.13
CD99	CD99	*0.78 ± 0.03	*0.64 ± 0.10	*0.59 ± 0.10	*0.39 ± 0.03
Meprin A subunit alpha	MEP1A	*1.91 ± 0.36	*2.02 ± 0.27	*2.32 ± 0.71	2.37 ± 0.96
Solute carrier family 15 member 1	SLC15A1	*1.56 ± 0.09	*1.68 ± 0.14	*1.73 ± 0.15	*1.81 ± 0.36
Sodium/glucose cotransporter 1	SLC5A1	*1.40 ± 0.16	*1.73 ± 0.10	*1.94 ± 0.37	*2.20 ± 0.28
Superoxide dismutase, mitochondrial	SOD2	*1.27 ± 0.11	*1.43 ± 0.14	*1.42 ± 0.07	*1.56 ± 0.01
Dual oxidase 2	DUOX2	*1.39 ± 0.18	*1.54 ± 0.32	*1.70 ± 0.29	*1.76 ± 0.19
Vitamin D-binding protein	GC	*1.56 ± 0.28	*1.65 ± 0.20	*1.86 ± 0.42	1.84 ± 0.85

### Effects of Aquamin^®^ on Proteins With Transporter Function

Table 2 also lists a number of proteins that are involved in electrolyte balance and water reabsorption from the colon. Down-regulation of several of these transporters occurs as a consequence of gastrointestinal inflammation and underlie the occurrence of diarrhea in UC and other colitis conditions (Yang et al., 1998; Gurney et al., 2017; Anbazhagan et al., 2018). Several of these proteins were increased, some substantially, with Aquamin^®^ (Table 2C).

**TABLE 2C:** Transporters.

		Aquamin			
Proteins	Genes	1.5 mM	2.1 mM	3.0 mM	4.5 mM
Sodium/hydrogen exchanger 1	NHE-1	1.22 ± 0.22	1.17 ± 0.35	1.29 ± 0.36	*1.35 ± 0.14
Sodium/hydrogen exchanger 2	NHE-2	1.11 ± 0.21	1.08 ± 0.28	1.19 ± 0.32	1.18 ± 0.33
Sodium/hydrogen exchanger 3	NHE-3	1.15 ± 0.10	1.29 ± 0.21	*1.40 ± 0.14	*1.24 ± 0.03
Na^(+)^/H^(+)^ exchange regulatory cofactor	PDZK1	1.21 ± 0.30	1.24 ± 0.37	1.24 ± 0.35	1.30 ± 0.36
Chloride anion exchanger	DRA	*1.77 ± 0.29	*1.91 ± 0.43	*1.89 ± 0.28	*2.10 ± 0.39
Sodium/potassium-transporting ATPase alpha-1	ATP1A1	1.36 ± 0.30	1.39 ± 0.26	*1.41 ± 0.25	1.52 ± 0.40
Sodium/potassium-transporting ATPase beta-1	ATP1B1	*1.46 ± 0.28	*1.52 ± 0.26	*1.58 ± 0.25	*1.73 ± 0.41
Sodium/potassium-transporting ATPase beta-3	ATP1B3	1.22 ± 0.36	1.27 ± 0.23	1.18 ± 0.23	1.27 ± 0.34

*The numerical values represent mean ± SD (fold-change of abundance ratio) as compared to the control, based on UC colonic tissue (n = 3 subjects). Each subject was assessed separately using TMT based differential proteomic expression and the data from the individual sets averaged together. False Discovery rate (FDR) for these proteins was <1%. Asterisks indicate statistical significance as compared to control at p < 0.05. HSPG2, heparan sulfate proteoglycan 2; CEACAM, carcinoembryonic antigen-related cell adhesion molecule; NRAM2, natural resistance-associated macrophage protein 2.*

### Unbiased Assessment of Proteins Up-Regulated or Down-Regulated With Multi-Mineral Intervention

The three proteomic TMT 6-plex experiments (one per each UC subject) identified 3932 unique proteins present across the 3 subjects with a <2% FDR. The proteins presented in Table 2 were chosen based on a known or suspected role in differentiation and barrier formation or in consideration of their relevance to the pathophysiology of inflammatory bowel disease. In parallel, we conducted an unbiased search of the database for all proteins up- or down-regulated by Aquamin^®^ treatment. The list of proteins that met the criteria of 1.8-fold increase or decrease across the three data sets (i.e., from each subject) with a <2% FDR are shown in Tables 3, 4 along with pathways linked to these proteins. Twenty-nine proteins were up-regulated in all three specimens (Table 3). Of interest, the majority of these unbiased moieties were proteins involved in differentiation and barrier formation – i.e., the same proteins presented in Table 2. There were 50 down-regulated proteins that met this criterion (Table 4). As can be seen from Table 4, many of these were involved in nucleotide metabolism, proliferation-related signaling and proliferation. For example, proliferating cell nuclear antigen (PCNA), proliferation marker protein Ki67, nucleophosmin and stathmin were significantly decreased by Aquamin^®^. Additionally, moieties (CD44, and CD99) involved in the lymphocyte and polymorphonuclear leukocytes trafficking and correlated positively with inflammatory bowel disease (IBD) disease activity, were also significantly decreased (Wittig et al., 1998; Brazil et al., 2010; Zhou et al., 2017). An overexpression of CD44 is also associated with unfavorable prognosis in colorectal cancer patients (Wang et al., 2019). Pathways associated with these up- and down-regulated proteins are presented in Tables 3B, 4B. Laminin interactions, fibronectin matrix formation and extracellular matrix organization were top three significantly up-regulated pathways (Table 3B). On the other hand, the top three most significant down-regulated pathways were unwinding of DNA, DNA strand elongation and rRNA processing in the nucleus and cytosol (Table 4B). As part of our investigation, we searched the proteomic database for putative stem cell markers including OLFM4, LGR5, LRIG1 and LRRC40. Of these, LGR5 and LRIG1 were not detected; LRRC40 was reduced from control by nearly 28% with Aquamin^®^ while OLFM-4 was slightly increased.

**TABLE 3A:** Up-regulated proteins: unbiased proteomic screen.

		Aquamin			
Proteins	Genes	1.5 mM	2.1 mM	3.0 mM	4.5 mM
Nuclear receptor 0B1^#^	NR0B1	2.59 ± 1.13	5.71 ± 3.91	4.69 ± 2.45	6.68 ± 4.11
CEACAM 1	CEACAM1	1.72 ± 0.52	5.25 ± 4.20	2.01 ± 1.12	1.80 ± 1.18
Alpha-2-HS-glycoprotein	AHSG	2.24 ± 0.87	2.39 ± 1.18	2.59 ± 1.69	2.48 ± 0.95
Cadherin-17	CDH17	*2.83 ± 0.22	*2.95 ± 0.60	*3.23 ± 0.67	*3.62 ± 0.70
Calcium-activated chloride channel regulator 4	CLCA4	*2.03 ± 0.28	*2.24 ± 0.51	*2.31 ± 0.27	*3.01 ± 0.74
Zinc transporter ZIP4	SLC39A4	*2.01 ± 0.55	*2.08 ± 0.46	*2.63 ± 0.77	*2.83 ± 0.96
Ly6/PLAUR domain-containing protein 8	LYPD8	*2.12 ± 0.22	*2.14 ± 0.50	*2.46 ± 0.40	*2.64 ± 0.93
Meprin A subunit alpha	MEP1A	*1.91 ± 0.36	*2.02 ± 0.27	*2.32 ± 0.71	2.37 ± 0.96
Natural resistance-associated macrophage protein 2	SLC11A2	*1.91 ± 0.42	*2.20 ± 0.43	*2.56 ± 0.36	*2.68 ± 0.54
Laminin subunit beta-1	LAMB1	*2.14 ± 0.70	*2.20 ± 0.48	*2.12 ± 0.56	*2.85 ± 0.42
Laminin subunit alpha-1	LAMA1	*2.00 ± 0.62	*2.15 ± 0.38	*2.00 ± 0.49	*2.59 ± 0.44
Integrin alpha-5	ITGA5	*1.69 ± 0.42	*1.80 ± 0.26	*2.21 ± 0.58	*2.27 ± 0.67
CEACAM 7	CEACAM7	*1.83 ± 0.32	*2.00 ± 0.08	*2.23 ± 0.37	*2.44 ± 0.50
Apolipoprotein A-I	APOA1	1.62 ± 0.61	*1.96 ± 0.46	*2.39 ± 0.28	*2.68 ± 0.30
CEACAM 5	CEACAM5	1.37 ± 0.29	*2.37 ± 0.50	1.81 ± 0.79	1.71 ± 0.82
Laminin subunit gamma-1	LAMC1	*2.03 ± 0.58	*2.07 ± 0.37	*2.01 ± 0.47	*2.68 ± 0.30
Desmoglein-2	DSG2	*2.07 ± 0.17	*2.10 ± 0.25	*2.28 ± 0.37	*2.36 ± 0.40
Fucose mutarotase	FUOM	*1.44 ± 0.13	*1.76 ± 0.25	*1.83 ± 0.19	*2.04 ± 0.58
Laminin subunit beta-2	LAMB2	*1.91 ± 0.49	*2.03 ± 0.34	*1.90 ± 0.30	*2.32 ± 0.30
Cyclic AMP-dependent transcription factor ATF-7^#^	ATF7	*1.37 ± 0.13	*2.25 ± 0.37	*1.76 ± 0.45	1.74 ± 0.57
CEACAM 6	CEACAM6	1.25 ± 0.35	*2.13 ± 0.35	1.50 ± 0.78	1.33 ± 0.80
Sodium/glucose cotransporter 1	SLC5A1	*1.40 ± 0.16	*1.73 ± 0.10	*1.94 ± 0.37	*2.20 ± 0.28
Nidogen-1	NID1	1.70 ± 0.49	*1.66 ± 0.37	*1.72 ± 0.37	*2.11 ± 0.28
Copper-transporting ATPase 2	ATP7B	1.39 ± 0.52	*1.62 ± 0.22	*1.94 ± 0.16	*2.28 ± 0.04
ENPP3	ENPP3	*1.41 ± 0.15	*1.60 ± 0.18	*1.81 ± 0.19	*2.04 ± 0.25
Aminopeptidase N	ANPEP	*1.91 ± 0.21	*2.01 ± 0.25	*1.85 ± 0.27	*2.00 ± 0.36
Beta-glucuronidase	GUSB	*1.29 ± 0.06	*1.51 ± 0.23	*1.51 ± 0.17	*2.00 ± 0.27
Hydroxymethylglutaryl-CoA synthase, mitochondrial	HMGCS2	*1.52 ± 0.12	*1.74 ± 0.23	*1.95 ± 0.22	*2.09 ± 0.18
Inter-alpha-trypsin inhibitor heavy chain H4	ITIH4	*1.46 ± 0.08	*1.78 ± 0.31	*1.79 ± 0.32	*1.98 ± 0.20

*The numerical values represent mean ± SD (fold-change of abundance ratio) as compared to the control, based on UC colonic tissue (n = 3 subjects). Each subject was assessed separately using TMT based differential proteomic expression and the data from the individual sets averaged together. False Discovery rate (FDR) for all the proteins was < 1% except for two proteins (^#^FDR < 2%). Values were ordered from maximum to minimum fold-change based on the maximum response with any of the Aquamin^®^ concentrations. To be included, values were up-regulated by at least 1.8-fold with at least one concentration of Aquamin^®^ as compared to control and common in all three subjects. Asterisks indicate statistical significance as compared to control at p < 0.05. CEACAM, Carcinoembryonic antigen-related cell adhesion molecule; ENPP3, ectonucleotide pyrophosphatase/phosphodiesterase family member 3.*

**TABLE 3B:** Pathways significantly activated based on up-regulated proteins.

Pathway name	pValue	FDR
Laminin interactions	2 × 10^–8^	2 × 10^–6^
Fibronectin matrix formation	1 × 10^–6^	4 × 10^–5^
Extracellular matrix organization	1 × 10^–6^	4 × 10^–5^
MET activates PTK2 signaling	1 × 10^–6^	4 × 10^–5^
MET promotes cell motility	5 × 10^–6^	1 × 10^–4^
Post-translational protein phosphorylation	1 × 10^–5^	2 × 10^–4^
Regulation of IGF transport and uptake by IGF Binding Proteins (IGFBPs)	2 × 10^–5^	3 × 10^–4^
Non-integrin membrane-ECM interactions	2 × 10^–5^	3 × 10^–4^
ECM proteoglycans	5 × 10^–5^	7 × 10^–4^
Signaling by MET	6 × 10^–5^	7 × 10^–4^
L1CAM interactions	3 × 10^–4^	3 × 10^–3^
Disorders of transmembrane transporters	1 × 10^–3^	0.01
Post-translational modification: synthesis of GPI-anchored proteins	2 × 10^–3^	0.01
Metal ion SLC transporters	2 × 10^–3^	0.01
MPS VII - Sly syndrome	3 × 10^–3^	0.01
Defective SLC39A4 causes acrodermatitis enteropathica, zinc-deficiency	3 × 10^–3^	0.01
Defective SLC11A2 causes hypochromic microcytic anemia	3 × 10^–3^	0.01
Defective SLC5A1 causes congenital glucose/galactose malabsorption	3 × 10^–3^	0.01
SLC transporter disorders	3 × 10^–3^	0.01
Cell surface interactions at the vascular wall	4 × 10^–3^	0.02
Platelet degranulation	5 × 10^–3^	0.02
Response to elevated platelet cytosolic Ca^2+^	6 × 10^–3^	0.02
Degradation of the extracellular matrix	8 × 10^–3^	0.02
Neutrophil degranulation	0.008	0.03
Post-translational protein modification	0.01	0.04
Intestinal hexose absorption	0.01	0.04
Transport of small molecules	0.01	0.04
HDL clearance	0.01	0.04
Intestinal absorption	0.01	0.04
Scavenging by Class B Receptors	0.02	0.05
GDP-fucose biosynthesis	0.02	0.05
HDL assembly	0.02	0.05
Nuclear Receptor transcription pathway	0.02	0.05
Transport of bile salts and organic acids, metal ions and amine compounds	0.02	0.05
RUNX2 regulates genes involved in cell migration	0.02	0.05
Chylomicron remodeling	0.03	0.05
Chylomicron assembly	0.03	0.05
Zinc influx into cells by the SLC39 gene family	0.03	0.05
HDL remodeling	0.03	0.05
Mucopolysaccharidoses	0.03	0.05
Apoptotic cleavage of cell adhesion proteins	0.03	0.05
SLC-mediated transmembrane transport	0.03	0.05
Hyaluronan uptake and degradation	0.03	0.05
Metabolism of proteins	0.03	0.05
Platelet activation, signaling and aggregation	0.03	0.05
PPARA activates gene expression	0.04	0.05
Signaling by Receptor Tyrosine Kinases	0.04	0.05
Regulation of lipid metabolism by Peroxisome proliferator-activated receptor α	0.04	0.05
Hyaluronan metabolism	0.04	0.05
Vitamin B5 (pantothenate) metabolism	0.04	0.05
Zinc transporters	0.04	0.05
ABC transporters in lipid homeostasis	0.05	0.05
Metabolism of Angiotensinogen to Angiotensins	0.05	0.05
Plasma lipoprotein assembly	0.05	0.05
Scavenging by Class A Receptors	0.05	0.05

*The pathway analysis report was generated by Reactome (v70) for species “Homo sapiens.” These significant data (with a p-value/FDR < 0.05) are based on the overrepresentation analysis (hypergeometric distribution).*

**TABLE 4A:** Down-regulated proteins: unbiased proteomic screen.

		Aquamin			
Proteins	Genes	1.5 mM	2.1 mM	3.0 mM	4.5 mM
Neurogranin	NRGN	*0.54 ± 0.05	*0.38 ± 0.16	*0.30 ± 0.13	*0.18 ± 0.09
Fibrinogen silencer-binding protein^#^	FSBP	*0.61 ± 0.09	*0.44 ± 0.10	*0.36 ± 0.03	*0.22 ± 0.13
Stathmin	STMN1	*0.58 ± 0.07	*0.47 ± 0.02	*0.37 ± 0.01	*0.29 ± 0.12
DNA replication licensing factor MCM3	MCM3	*0.70 ± 0.07	*0.57 ± 0.06	*0.44 ± 0.07	*0.34 ± 0.13
Nucleolar GTP-binding protein 2	GNL2	*0.59 ± 0.01	*0.64 ± 0.15	*0.55 ± 0.04	*0.30 ± 0.08
Heterogeneous nuclear ribonucleoprotein A0	HNRNPA0	*0.71 ± 0.09	*0.55 ± 0.19	*0.49 ± 0.20	*0.39 ± 0.16
Pumilio homolog 3^#^	PUM3	0.78 ± 0.25	*0.70 ± 0.06	*0.46 ± 0.14	*0.33 ± 0.19
U3 small nucleolar RNA-interacting protein 2	RRP9	*0.62 ± 0.03	*0.51 ± 0.06	*0.38 ± 0.01	*0.32 ± 0.10
Ribonucleoside-diphosphate reductase	RRM1	*0.68 ± 0.06	*0.56 ± 0.21	*0.46 ± 0.14	*0.35 ± 0.15
Inosine-5′-monophosphate dehydrogenase 2	IMPDH2	*0.63 ± 0.03	*0.56 ± 0.13	*0.43 ± 0.11	*0.35 ± 0.12
DNA replication licensing factor MCM2	MCM2	*0.70 ± 0.06	*0.62 ± 0.16	*0.47 ± 0.15	*0.32 ± 0.11
Proliferating cell nuclear antigen	PCNA	*0.65 ± 0.01	*0.59 ± 0.08	*0.45 ± 0.05	*0.36 ± 0.10
Proliferation marker protein Ki67	MKI67	*0.58 ± 0.08	*0.48 ± 0.11	*0.39 ± 0.13	*0.31 ± 0.08
Nucleolar and coiled-body phosphoprotein 1	NOLC1	*0.63 ± 0.05	*0.53 ± 0.06	*0.44 ± 0.06	*0.37 ± 0.10
Nucleophosmin	NPM1	*0.59 ± 0.06	*0.55 ± 0.06	*0.43 ± 0.04	*0.35 ± 0.09
Nuclear autoantigenic sperm protein	NASP	*0.66 ± 0.07	*0.57 ± 0.03	*0.45 ± 0.03	*0.37 ± 0.11
Serine/arginine-rich splicing factor 6	SRSF6	*0.67 ± 0.15	*0.56 ± 0.11	*0.47 ± 0.10	*0.37 ± 0.10
Serine/threonine-protein kinase VRK1	VRK1	*0.70 ± 0.07	*0.61 ± 0.11	*0.52 ± 0.11	*0.41 ± 0.12
Protein RRP5 homolog	PDCD11	*0.72 ± 0.03	*0.54 ± 0.12	*0.44 ± 0.15	*0.41 ± 0.06
Nucleolin	NCL	*0.70 ± 0.09	*0.57 ± 0.02	*0.48 ± 0.04	*0.41 ± 0.11
PC4 and SFRS1-interacting protein	PSIP1	*0.72 ± 0.10	*0.56 ± 0.05	*0.48 ± 0.05	*0.41 ± 0.12
Non-histone chromosomal protein HMG-17	HMGN2	*0.73 ± 0.05	*0.55 ± 0.18	*0.49 ± 0.16	*0.43 ± 0.13
DNA replication licensing factor MCM7	MCM7	*0.77 ± 0.08	0.74 ± 0.23	*0.56 ± 0.23	*0.41 ± 0.12
Heterogeneous nuclear ribonucleoprotein D-like	HNRNPDL	*0.69 ± 0.06	*0.59 ± 0.02	*0.50 ± 0.04	*0.42 ± 0.11
SWI/SNF complex subunit SMARCC1	SMARCC1	*0.69 ± 0.08	*0.54 ± 0.07	*0.48 ± 0.04	*0.41 ± 0.13
NOP2	NOP2	*0.74 ± 0.06	*0.60 ± 0.09	*0.51 ± 0.06	*0.43 ± 0.12
Nucleolar RNA helicase 2	DDX21	*0.63 ± 0.07	*0.52 ± 0.12	*0.43 ± 0.06	*0.34 ± 0.04
PIN4	PIN4	*0.66 ± 0.09	*0.61 ± 0.07	*0.51 ± 0.09	*0.47 ± 0.14
DNA replication licensing factor MCM5	MCM5	*0.70 ± 0.10	*0.57 ± 0.18	*0.49 ± 0.16	*0.40 ± 0.13
CD44 antigen	CD44	*0.61 ± 0.01	*0.58 ± 0.12	*0.48 ± 0.12	*0.40 ± 0.09
Coiled-coil domain-containing protein 86	CCDC86	*0.69 ± 0.02	*0.52 ± 0.13	*0.45 ± 0.10	*0.42 ± 0.11
Gem-associated protein 5	GEMIN5	*0.82 ± 0.11	*0.65 ± 0.13	*0.53 ± 0.16	*0.47 ± 0.11
Zinc finger C2HC domain-containing protein 1A	ZC2HC1A	*0.54 ± 0.17	*0.50 ± 0.09	*0.65 ± 0.18	*0.54 ± 0.11
Translation machinery-associated protein 7	TMA7	*0.63 ± 0.07	*0.57 ± 0.10	*0.49 ± 0.06	*0.44 ± 0.09
DNA replication licensing factor MCM4	MCM4	*0.70 ± 0.02	*0.67 ± 0.09	*0.52 ± 0.07	*0.43 ± 0.10
Ras GTPase-activating protein-binding protein 1	G3BP1	*0.75 ± 0.07	*0.61 ± 0.10	*0.55 ± 0.09	*0.47 ± 0.10
CD99 antigen	CD99	*0.78 ± 0.03	*0.64 ± 0.10	*0.59 ± 0.10	*0.39 ± 0.04
NHP2-like protein 1	SNU13	*0.69 ± 0.04	*0.57 ± 0.08	*0.53 ± 0.08	*0.47 ± 0.11
Receptor-type tyrosine-protein phosphatase F	PTPRF	*0.56 ± 0.09	*0.55 ± 0.11	*0.50 ± 0.08	*0.45 ± 0.07
Putative RNA-binding protein Luc7-like 1	LUC7L	*0.69 ± 0.07	*0.72 ± 0.11	*0.58 ± 0.15	*0.47 ± 0.07
HEAT repeat-containing protein 1	HEATR1	*0.69 ± 0.17	*0.64 ± 0.08	*0.55 ± 0.07	*0.43 ± 0.03
U6 snRNA-associated Sm-like protein LSm5	LSM5	*0.77 ± 0.09	*0.61 ± 0.12	*0.56 ± 0.07	*0.48 ± 0.10
Importin subunit alpha-1	KPNA2	*0.70 ± 0.08	*0.66 ± 0.11	*0.57 ± 0.05	*0.48 ± 0.04
Palmitoyl-protein thioesterase 1	PPT1	*0.66 ± 0.03	*0.63 ± 0.05	*0.60 ± 0.03	*0.49 ± 0.05
Leydig cell tumor 10 kDa protein homolog	C19orf53	*0.67 ± 0.01	*0.63 ± 0.02	*0.56 ± 0.02	*0.49 ± 0.07
Ribosome biogenesis protein WDR12	WDR12	*0.77 ± 0.11	*0.67 ± 0.10	*0.58 ± 0.04	*0.47 ± 0.05
Transformer-2 protein homolog beta	TRA2B	0.85 ± 0.24	*0.56 ± 0.07	*0.53 ± 0.04	*0.48 ± 0.06
Ribosome biogenesis protein BMS1 homolog	BMS1	0.92 ± 0.32	0.79 ± 0.34	*0.61 ± 0.15	*0.49 ± 0.04
Drebrin	DBN1	*0.70 ± 0.08	*0.60 ± 0.05	*0.59 ± 0.02	*0.53 ± 0.09
BRCA2 and CDKN1A-interacting protein	BCCIP	*0.70 ± 0.06	*0.68 ± 0.07	*0.57 ± 0.03	*0.52 ± 0.06
Ubiquitin-associated protein 2-like	UBAP2L	*0.72 ± 0.05	*0.62 ± 0.07	*0.57 ± 0.04	*0.55 ± 0.07

*The numerical values represent mean ± SD (fold-change of abundance ratio) as compared to the control, based on UC colonic tissue (n = 3 subjects). Each subject was assessed separately using TMT based differential proteomic expression and the data from the individual sets averaged together. False Discovery rate (FDR) for all the proteins was <1% except for two proteins (^#^FDR < 2%). Values were ordered from minimum to maximum fold-change based on the minimum response with any of the Aquamin^®^ concentrations. To be included, values were down-regulated by at least 1.8-fold with at least one concentration of Aquamin^®^ as compared to control and common in all three subjects. Asterisks indicate statistical significance as compared to control at p < 0.05. NOP2, Probable 28S rRNA [cytosine(4447)-C(5)]-methyltransferase; PIN4, peptidyl-prolyl cis-trans isomerase NIMA-interacting 4.*

**TABLE 4B:** Pathways significantly activated based on down-regulated proteins.

Pathway name	pValue	FDR
Unwinding of DNA	3 × 10^–9^	3 × 10^–7^
DNA strand elongation	1 × 10^–8^	5 × 10^–7^
rRNA processing in the nucleus and cytosol	2 × 10^–7^	9 × 10^–6^
rRNA processing	3 × 10^–7^	1 × 10^–5^
rRNA modification in the nucleus and cytosol	3 × 10^–7^	1 × 10^–5^
Activation of the pre-replicative complex	5 × 10^–7^	1 × 10^–5^
Activation of ATR in response to replication stress	1 × 10^–6^	2 × 10^–5^
Metabolism of RNA	1 × 10^–6^	2 × 10^–5^
Major pathway of rRNA processing in the nucleolus and cytosol	2 × 10^–6^	2 × 10^–6^
Assembly of the pre-replicative complex	2 × 10^–5^	2 × 10^–4^
Synthesis of DNA	2 × 10^–5^	2 × 10^–4^
Orc1 removal from chromatin	2 × 10^–5^	2 × 10^–4^
DNA Replication	2 × 10^–5^	3 × 10^–4^
G1/S Transition	3 × 10^–5^	3 × 10^–4^
DNA Replication Pre-Initiation	4 × 10^–5^	4 × 10^–4^
Switching of origins to a post-replicative state	6 × 10^–5^	5 × 10^–4^
Mitotic G1-G1/S phases	6 × 10^–5^	5 × 10^–4^
S Phase	9 × 10^–5^	7 × 10^–4^
TFAP2A as a transcriptional repressor during retinoic acid-induced differentiation	3 × 10^–4^	3 × 10^–4^
G2/M Checkpoints	6 × 10^–4^	4 × 10^–3^
mRNA Splicing - Major Pathway	1 × 10^–3^	0.01
mRNA Splicing	2 × 10^–3^	0.01
Processing of Capped Intron-Containing Pre-mRNA	0.01	0.04
Cell Cycle	0.01	0.04
Cell Cycle Checkpoints	0.01	0.05
Cell Cycle, Mitotic	0.01	0.05

*The pathway analysis report was generated by Reactome (v70) for species “Homo sapiens.” These significant data (with a p value/FDR < 0.05) are based on the overrepresentation analysis (hypergeometric distribution).*

**FIGURE 4:**
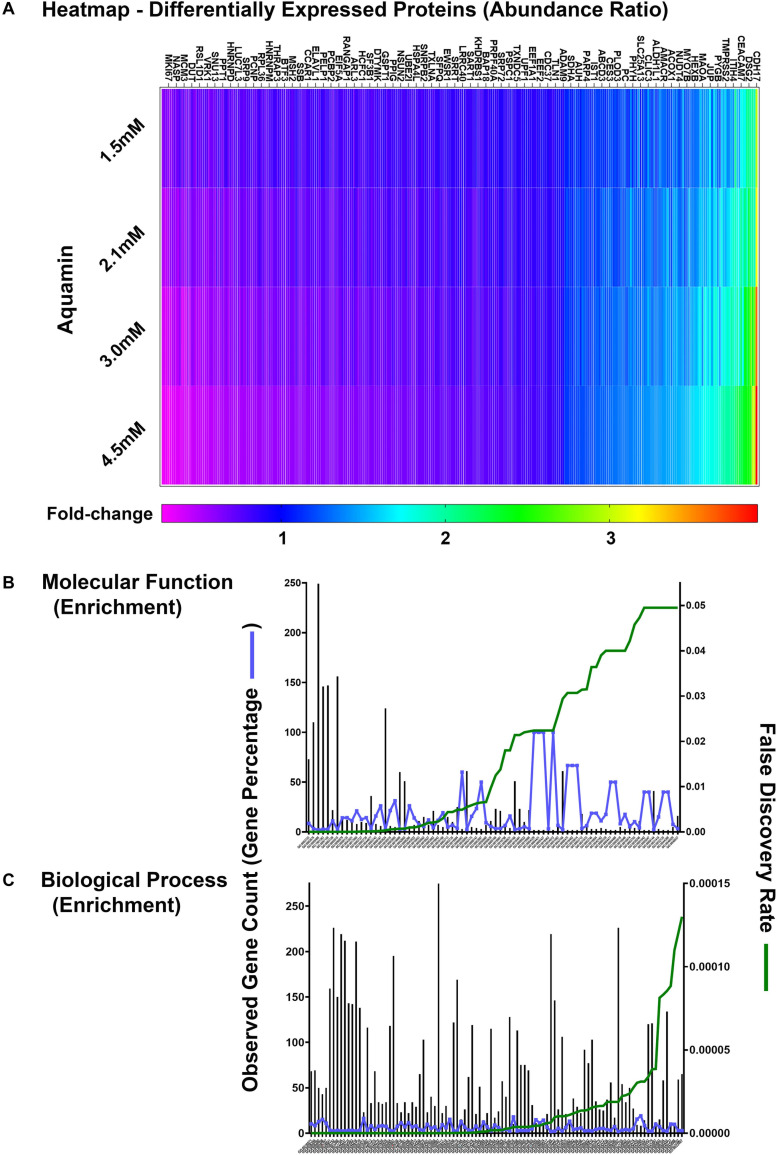
Quantitative proteomics analysis of ulcerative colitis tissue derived colonoids. **(A)** Heatmap of the 308 differentially expressed proteins that are significantly significant (*p* < 0.05) across all subjects (*n* = 3) and all culture conditions. **(A)** Complete list of these proteins are presented in Supplementary Table 5. Significantly enriched GO molecular functions **(B)** and GO biological processes **(C)** involving these proteins are shown. STRING-database (v11) was used for these enrichment analyses. For these graphs, observed genes (bars) and percentage of these genes as compared to the all involved genes (blue line) are plotted on the left *y*-axis. While false discovery rate is plotted on the right *y*-axis (green line). The GO annotation – molecular functions (78 functions) and biological processes (230 processes) are placed on the *x*-axis and listed in Supplementary Table 6.

Proteomic landscape was used to scale subject to subject variability and treatment response (Supplementary Figure 3). This figure displays the number of up- and down-regulated proteins across the three data sets at each of four levels of Aquamin^®^. The Venn plots in the upper panel show overlap at each Aquamin^®^ level separately among three subjects, and the plot in the lower level merges common proteins at all Aquamin^®^ concentrations and the three data sets. Supplementary Table 4 identifies individual proteins common at different levels of Aquamin^®^ across three subjects (shown in the Supplementary Figure 3). Lastly, Supplementary Table 5 presents a comprehensive list of proteins (308 proteins in all and up- or down-regulated) that met the criterion of being significant with a *p*-value of 0.05 or less across all conditions of Aquamin^®^ as compared to control (regardless of fold-change). These 308 proteins can be visualized as a heatmap (Figure 4A), which clearly shows a dose response trend from lower to higher concentration. There were 78 GO molecular functions and 330 GO biological processes affected through the interaction of these 308 proteins (Supplementary Table 6) – curated by STRING database. Figures 4B,C present all molecular functions and top 100 biological processes known to be affected by the involvement of these proteins.

### Cytokine Profile in Control and Mineral-Treated Colonoids

In a final set of experiments, we assessed 2-day culture fluids obtained from control and Aquamin^®^-treated colonoid cultures at day-14 (immediately prior to harvest) for levels of pro-inflammatory cytokines (TNF-α, IL-1β, IL6, IL-8, and MCP-1). While levels of TNF-α, IL-1β, IL6 and MCP-1 were near or below quantifiable limits, substantial amounts of IL-8 were detected in all culture fluids. The average level of IL-8 in control specimens was 410 ± 193 pg/mL (*n* = 7) and this was reduced to 362 ± 89 pg/mL and 344 ± 213 pg/mL in specimens treated with Aquamin^®^ (providing 1.5 and 2.1 mM calcium, respectively). For comparison, the level of IL-8 seen in colonoids initiated with tissue from healthy control subjects [available from our previous study (Attili et al., 2019) was 268 ± 103 pg/mL (*n* = 3)].

## Discussion

To test the beneficial role of Aquamin^®^ minerals in UC patient samples, we carried out the study described here using a novel 3D (three-dimensional) organoid culture technology: colonoid culture. This technology allowed us to obtain biopsy material from the human colonic mucosa and expand the tissue in culture. The advantage of this technology is that it provides a culture system that preserves the characteristic features of human colon tissue *in vivo*, yet allows for manipulation as readily as other simpler, *in vitro* culture systems (Dame et al., 2018; McClintock et al., 2018, 2020; Tsai et al., 2018; Attili et al., 2019). Going forward, this culture system will allow us to conduct an *ex vivo* trial using study participants’ tissue in parallel with an interventional trial. The long-term *in vivo* trial will take years to finish and then take additional time to analyze the data. The *ex vivo* approach may provide a snapshot of a subject well before the completion of the trial. We used a similar approach in our recently finished trial (clinicaltrials.gov; NCT02647671) in healthy subjects at risk for colorectal cancer (Attili et al., 2019; Aslam et al., 2020).

The colonic barrier is compromised in UC patients (Salim Sa and Söderholm, 2011; Antoni et al., 2014; Vivinus-Nebot et al., 2014; Lee et al., 2018), but whether this is simply the result of inflammatory injury to the epithelial lining, or whether pre-existing barrier weakness is a contributor to disease susceptibility has not been fully resolved. Regardless, it seems reasonable to suggest that strengthening the mucosal barrier could be of value. At the very least, an intact barrier would limit the permeation of both soluble and particulate pro-inflammatory moieties from the gut into the interstitium. Improvement in the barrier, especially, if it involved the basement membrane, might also retard passage of inflammatory cells into the epithelium to, presumably, reduce epithelial cell injury. The potential to improve the colonic barrier, as much as anything, drives the search for natural products and other adjuvant therapies for use in individuals with UC. Such therapies are unlikely to replace the potent anti-inflammatory drugs used in the treatment of acute disease flare-ups. Nonetheless, improvement in barrier function in UC patients who are in remission may be of value in so far as this could help maintain the state of remission and reduce symptomatology during this period. The findings presented in this manuscript suggest the importance of adequate mineral intake in this regard.

Here we demonstrate that treatment of UC tissue in colonoid culture with Aquamin^®^, a natural product consisting of calcium and magnesium along with 72 additional trace elements, up-regulates multiple proteins that contribute to the formation of a healthy barrier in the colon. Among these are proteins that comprise the primary cell-cell adhesion complexes – tight junctions, adherens junctions, and especially desmosomes. Also up-regulated are major non-collagenous components of the basement membrane along with hemidesmosomal components. Factors present on the apical surface (mucins, trefoils and CEACAMs) are also up-regulated.

Which of the multiple changes observed here are most important to improved barrier formation remains to be determined. While most studies have focused on tight junctions and on desmosomes (Schmitz et al., 1999; Zeissig et al., 2007; Kowalczyk and Green, 2013), other cellular and extracellular structures also contribute. Of these, the basement membrane may be the most important (Strater et al., 1996; Ferrell et al., 2011; Vllasaliu et al., 2014; Pozzi et al., 2017). The basement membrane supports barrier function in several important ways. The basement membrane inhibits bacterial crossing from the colonic stream into the interstitium and limits leukocyte trafficking from the circulation into the colonic epithelium. Additionally, the basement membrane is rich in anionic substances (heparin sulfate proteoglycans) – providing capacity to trap cationic moieties and regulate their permeation. Perhaps most importantly, the colonic wall consists of a single layer of epithelial cells and each cell is attached to the basement membrane. When cells detach from this substratum, they rapidly undergo apoptosis. With that, all barrier function is compromised. Thus, any compromise of the basement membrane could be impactful. Of interest in this regard, a loss of laminin in the colonic wall of individuals with UC has been noted (Schmehl et al., 2000; De Arcangelis et al., 2017). Also of interest, past studies have shown that disruption of the basement membrane or interference with epithelial cell binding to laminin (the major non-collagenous glycoprotein component of the basement membrane) results in widespread colitis in mice (Spenle et al., 2014).

Proteins expressed at the apical surface (mucins and trefoils) also have a role to play (Hensel et al., 2014). Mucins secreted by goblet cells are carbohydrate-rich molecules (Pullan et al., 1994). Trefoils are a family of proteins that interact with mucins and help organize the mucins into a viscous mucous network to trap bacteria and other particulates and to prevent their reaching the cell surface. Disruption of this network has been linked to inflammatory bowel disease (Loncar et al., 2003; Taupin and Podolsky, 2003; Aamann et al., 2014).

In addition to structural components of the barrier, a number of peptides with known anti-microbial activity were induced in response to the multi-mineral intervention (Table 2). These too could be considered part of the barrier, as could the bacterial film present on the colonocyte surface and bacteria present in the fecal stream (Schirmer et al., 2018). When a healthy microbial community is present, it limits the overgrowth of potentially harmful microbes. In part, this occurs via the healthy bacteria simply occupying a niche that could otherwise be colonized by pathogens. At the same time, the healthy microbial population can contribute to the barrier through elaboration of beneficial metabolites (e.g., short-chain fatty acids [SCFA]) rather than toxic bile acids. Previous studies have demonstrated microbial alterations in association with UC (Yang and Jobin, 2017) and have suggested that the make-up of the gastrointestinal microbial population can promote disease. While colonoid culture does not provide a ready-means to assess changes in the gut microbial community, we have recently completed a 90-day interventional trial with Aquamin^®^ in healthy human subjects (clinicaltrials.gov; NCT02647671). In the interventional trial, Aquamin^®^ ingestion has been found to alter the colonic microbial profile and, concomitantly, to decrease bile acids levels and increase the level of the major SCFA, acetate, as compared to placebo (or calcium alone) (Aslam et al., 2020). While the trial was conducted with healthy volunteers (at risk for colon cancer), a similar 180-days study in patients with UC in remission has just begun (clinicaltrials.gov; NCT03869905).

Along with anti-microbial peptides, our proteomic screen identified Aquamin^®^-induced changes in a number of proteins linked to gut inflammation. Pro-inflammatory elements were uniformly down-regulated while moieties with anti-inflammatory, anti-oxidant and anti-apoptotic activity were increased. It should be noted in regard to the inflammation-modulating moieties that these were detected in the colonic tissue itself, where cellular injury occurs. Of the secreted moieties related to inflammation, only IL-8 was detected in large amounts; it was decreased with Aquamin^®^. IL-8 has been correlated with IBD and mucosal inflammation (Daig et al., 1996). It should also be noted that several of the microbial and inflammation-modulating proteins responsive to intervention in UC colonoids were not detected at the threshold level in colonoids derived from healthy subjects’ tissue (Attili et al., 2019).

Finally, the proteomic assessment demonstrated that not only was there up-regulation of proteins that mediate the intercellular barrier, but that a number of proteins (i.e., transporters) that regulate electrolyte passage through the cell were also increased in response to Aquamin^®^. Reduced expression of several of these moieties is seen in UC as well as in experimental models of colitis (Yang et al., 1998; Gurney et al., 2017; Anbazhagan et al., 2018). Up-regulation with Aquamin^®^ could serve to normalize electrolyte transport (critical to water resorption) to reduce diarrhea, one of the most pervasive symptoms in UC as well as in other conditions of the gastrointestinal tract.

Our unbiased proteomic analysis showed that many of the down-regulated proteins were related to proliferation. Consistent with this, a reduction in Ki67 expression with intervention was seen by immunostaining. It could be argued that suppression of epithelial cell proliferation in the context of a colonic ulcer might be an unwanted consequence of intervention. While this possibility needs to be acknowledged, it is unlikely that effects on proliferation would counteract the beneficial activities related to improved barrier formation and reduced inflammation. Hyperplastic epithelial growth is part of the wound-healing process, including in the colon (Lyons et al., 2018), but its continued presence is a reflection of chronic wound formation. Suppression of hyperplastic growth in the epithelium (occurring as a normal component of the wound-healing process) is, ultimately, necessary. Furthermore, colonic hyperplasia/dysplasia is prognostically related to cancer development in UC patients (Spenle et al., 2014; Osone et al., 2019). A recent study has demonstrated that the accumulation of a protein referred to as Stathmin (STMN1) in UC lesions is associated with neoplasia (Osone et al., 2019). STMN1 was significantly down-regulated by Aquamin^®^ (up to 3.5 fold-reduction; Table 4). The growth regulatory moieties, e.g., proliferating cell nuclear antigen, proliferation marker protein Ki67 and nucleophosmin were also significantly down-regulated in response to Aquamin^®^. Thus, proliferation-regulating effects of Aquamin^®^ might contribute to reduced cancer development in UC patients. Ultimately, these complex questions will need to be addressed experimentally, and this is beyond the scope of the current work.

## Conclusion (and Implications)

**FIGURE 5:**
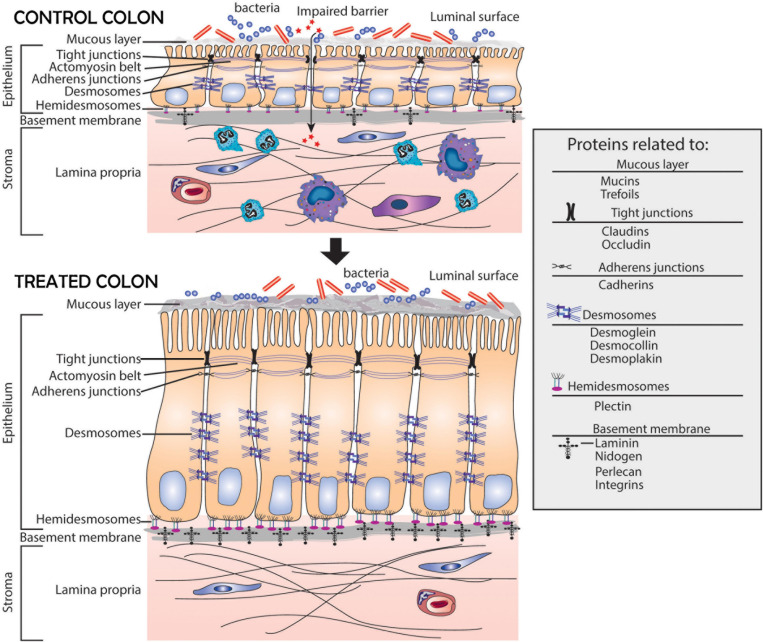
Schematic representation of the colonic mucosa in UC-derived colonoid culture and structural changes due to intervention with Aquamin^®^. Tight junctions are observed at the apical surface between adjacent cells in both control and treated colonoids; there is little observable difference between the two. Desmosomes (shown along the lateral surface between cells) are increased in response to treatment. This should support increased tissue strength. Additional changes resulting from Aquamin^®^ intervention include an increase in the non-collagenous components of the basement membrane and an increase in hemidesmosomal proteins. These changes should promote improved cell-matrix adhesion. Increased mucin and trefoil levels, leading to a thicker mucous layer at the luminal surface, should contribute to more efficient trapping of bacteria. In aggregate, these changes should provide for improved barrier function and may help mitigate colonic inflammation.

A schematic representation of the colonic mucosa in UC-derived colonoid culture is presented in Figure 5. It highlights structural changes in the barrier occurring in response to the multi-mineral intervention. Based on the results presented here, we hypothesize greater tissue strength, improved cell-basement membrane interaction and a thicker mucous layer above the apical surface. These changes along with the increased elaboration of anti-microbial and anti-inflammatory proteins should, ultimately, be beneficial to individuals with UC in remission.

A majority of individuals in Western society do not reach the recommended daily intake for calcium, according to U.S. Department of Agriculture 2015 – 2020 Dietary Guidelines for Americans (U.S. Department of Health and Human Services and U.S, 2015), and this deficiency contributes to multiple chronic diseases (Peterlik and Cross, 2009; Peterlik et al., 2009). Magnesium-deficiency is also common (Lennie et al., 2018). While there are no intake recommendations for many of the other potentially important trace elements in Aquamin^®^, deficiencies likely exist for those elements that are nutritionally associated with calcium or magnesium. While roles for calcium and magnesium in the prevention of chronic disease are at least partially understood, what role micronutrient deficiencies – including those involving minerals – play in chronic or long-latency diseases is only beginning to get attention (Lennie et al., 2018; Swaminath et al., 2019). To the extent that multi-mineral deficiencies are common, the findings presented here allow us to hypothesize that such deficiencies might contribute to the weakening of the colonic barrier in many individuals, including those with UC. Obtaining the proper intake of minerals, either through a healthful eating pattern or through the use of an appropriate multi-mineral supplement, should be beneficial.

Whether the multi-mineral product investigated here (Aquamin^®^) or some other mineral formulation will, ultimately, provide benefit to individuals with UC and whether the intervention will prove safe and tolerable needs to be established through precisely controlled clinical studies. While the necessary studies have not yet been conducted, we have just begun a pilot-phase trial (clinicaltrials.gov; NCT03869905) with UC patients in remission to address safety and tolerability concerns, and to begin generating efficacy data with Aquamin^®^.

## Data Availability Statement

The raw data supporting the conclusions of this article will be made available by the authors, without undue reservation. The raw mass spectrometry proteomics data is available on ProteomeXchange Consortium (PRIDE partner repository) – identifier PXD020244.

## Ethics Statement

The studies involving human participants were reviewed and approved by Institutional Review Board (IRBMED) at the University of Michigan. The patients/participants provided their written informed consent to participate in this study.

## Author Contributions

All the authors have contributed toward this project to claim authorship according to ICMJE criteria, involved in data acquisition and data analysis or data interpretation, contributed to manuscript revision, read, and approved the submitted version. MA, SM, DT, and JV contributed to the conception and design of the study. DT collected all the colon biopsies. MA performed the statistical analysis. MA and JV wrote the first draft of the manuscript. SM, DA, MD, and DT wrote sections of the manuscript.

## Conflict of Interest

The authors declare that the research was conducted in the absence of any commercial or financial relationships that could be construed as a potential conflict of interest.
