# Supplementary material for: Ulcerative Colitis-Derived Colonoid Culture: A Multi-Mineral-Approach to Improve Barrier Protein Expression
Source: Front Cell Dev Biol. 2020 Nov 23;8:577221. doi: 10.3389/fcell.2020.577221 (PMC7719760; doi:10.3389/fcell.2020.577221)
Supplement: Supplementary file 4 [file Presentation_1.pdf]

SUPPLEMENTAL MATERIAL (Complete list)

Supplementary Table 1.	Mineral composition of Aquamin®
Supplementary Table 2.	Antibody related information
Supplementary Table 3.	Statistical Analysis - *p* Value evaluation by GraphPad Prism 8.3 for data presented in Figure 1, 2 and 3.
Supplementary Table 4.	A list of altered (and common) proteins in response to different concentrations of Aquamin® at 1.8 fold-change.
Supplementary Table 5.	A comprehensive list of altered proteins with Aquamin® (*p* value <0.05 across all conditions and subjects)
Supplementary Table 6.	A) A list of GO Molecular Functions assessed by STRING-db<br>B) A list of GO Biological Processes assessed by STRING-db<br>—related to proteins significantly altered with Aquamin® and presented in Supplement Figure 2 and Supplement Table 5)
Supplementary Figure 1.	UC colonoid appearance: Phase-contrast, Histological features (H&E) and CK20 expression.
Supplementary Figure 2.	Protein-protein interactions—STRING-database (for proteins listed in Table 2).
Supplementary Figure 3.	Relationship of protein changes comparing proteomic signature of three UC patients across all culture conditions.
Supplemental File 1.	Additional citations - differentiation / barrier related proteins presented in the Table 2.

Supplemental File 1. Additional References (Relevant to the moieties presented in the Table 2).

Antimicrobial peptides

- Arijs I, De Hertogh G, Lemaire K, et al. Mucosal gene expression of antimicrobial peptides in inflammatory bowel disease before and after first infliximab treatment. *PloS one*. 2009;4:e7984.
- Okumura R, Kurakawa T, Nakano T, et al. Lypd8 promotes the segregation of flagellated microbiota and colonic epithelia. *Nature*. 2016;532:117.
- Okumura R, Takeda K. Maintenance of intestinal homeostasis by mucosal barriers. *Inflammation and regeneration*. 2018;38:5.
- Cellier MF, Courville P, Campion C. Nramp1 phagocyte intracellular metal withdrawal defense. *Microbes and infection*. 2007;9:1662-70.
- Orsi N. The antimicrobial activity of lactoferrin: current status and perspectives. *Biometals*. 2004;17:189-96.
- Man SM, Karki R, Kanneganti TD. DNA-sensing inflammasomes: regulation of bacterial host defense and the gut microbiota. *Pathogens and disease*. 2016;74(4).
- Jasir A, Kasprzykowski F, Kasprzykowska R, et al. New antimicrobial cystatin C-based peptide active against gram-positive bacterial pathogens, including methicillin-resistant Staphylococcus aureus and multiresistant coagulase-negative staphylococci. *Apmis*. 2003;111:1004-10.
- Pulkkinen V, Bruce S, Rintahaka J, et al. ELMOD2, a candidate gene for idiopathic pulmonary fibrosis, regulates antiviral responses. *The FASEB Journal.* 2010;24:1167-77.

Inflammation

- Mitsuyama K, Toyonaga A, Sasaki E, et al. IL-8 as an important chemoattractant for neutrophils in ulcerative colitis and Crohn's disease. *Clin Exp Immunol*. 1994;96:432–436.
- Zietek T, Rath E. Inflammation Meets Metabolic Disease: Gut Feeling Mediated by GLP-1. *Front Immunol*. 2016;7:154.
- Plevy S, Silverberg MS, Lockton S, et al. Combined serological, genetic, and inflammatory markers differentiate non-IBD, Crohn's disease, and ulcerative colitis patients. *Inflamm Bowel Dis.* 2013;19:1139–1148.
- Palone F, Vitali R, Cucchiara S, et al. Fecal HMGB1 reveals microscopic inflammation in adult and pediatric patients with inflammatory bowel disease in clinical and endoscopic remission. *Inflammatory bowel diseases*. 2016;22:2886-93.
- Zhu S, Ding S, Wang P, et al. Nlrp9b inflammasome restricts rotavirus infection in intestinal epithelial cells. *Nature*. 2017;546:667.

Oxidative stress

- Guo F, He H, Fu ZC, et al. Adipocyte-derived PAMM suppresses macrophage inflammation by inhibiting MAPK signalling. *Biochem J*. 2015;472:309–318.
- Geiszt M, Witta J, Baffi J, et al. Dual oxidases represent novel hydrogen peroxide sources supporting mucosal surface host defense. *The FASEB journal*. 2003;17:1502-4.
- Guan G, Lan S. Implications of Antioxidant Systems in Inflammatory Bowel Disease. *Biomed Res Int*. 2018;1290179. doi:10.1155/2018/1290179

Apolipoprotein A-I

- Meriwether D, Sulaiman D, Volpe C, et al. Apolipoprotein AI mimetics mitigate intestinal inflammation in COX2-dependent inflammatory bowel disease model. *J Clin Invest*. 2019;130:3670-3685.

ZO / Myosin

- Cordenonsi M, D'Atri F, Hammar E, et al. Cingulin contains globular and coiled-coil domains and interacts with ZO-1, ZO-2, ZO-3, and myosin. *J Cell Biol*. 1999;147:1569–1582.

- Liu KC, Cheney RE. Myosins in cell junctions. *Bioarchitecture*. 2012;2:158–170.
- Vicente-Manzanares M, Ma X, Adelstein RS, et al. Non-muscle myosin II takes centre stage in cell adhesion and migration. *Nature reviews Molecular cell biology*. 2009;10:778.
