# Supplementary material for: Ulcerative Colitis-Derived Colonoid Culture: A Multi-Mineral-Approach to Improve Barrier Protein Expression
Source: Front Cell Dev Biol. 2020 Nov 23;8:577221. doi: 10.3389/fcell.2020.577221 (PMC7719760; doi:10.3389/fcell.2020.577221)
Supplement: Supplementary file 5 [file Presentation_2.pdf]

SUPPLEMENTARY FIGURE LEGEND

Supplementary Figure 1. UC colonoid appearance. Phase-contrast microscopy (A): At the end of the incubation period, intact colonoids were examined by phase-contrast microscopy. Colonoids were present as thick-walled structures with few surface buds. A wide range of sizes and shapes were seen under all conditions. The zoomed-out (top) and zoomed-in (below) appearance of colonoids under a phase-contrast microscope. Bar=400 (top), Bar=200µm (bottom). Histological features (B): At the end of the incubation period, colonoids were examined by light microscopy after staining with hematoxylin and eosin. Under low-calcium conditions (Control), the colonoids were found to be crypts of varying size with a single layer of epithelial cells surrounding a central lumen. Tiny crypts (with as few as 20 cells in cross section) were seen. In the presence of Aquamin®, larger crypts made up of columnar epithelial cells surrounding a large, often irregular-shaped lumen were seen. Goblet cells were apparent. Lower magnification (top) and higher magnification (below)
Bars=100µm. CK20 expression (C): Immunohistology revealed high-expression of CK20 under all conditions. Lower magnification (top) and higher magnification (below). Bars=100µm.

Supplementary Figure 2. Cell-cell and cell-matrix adhesion protein-protein interactions—STRING-database (v11). These proteins are listed in Table 2. There are 3-4 distinct clusters of proteins and strong interactions or associations can be seen with thick connecting lines**.**

Supplementary Figure 3. Protein distribution with differing Aquamin® levels across three subjects. At the end of the incubation period, lysates from all the conditions (and three subjects) were prepared for proteomic analysis. **Upper panel**: Venn plots (generated by BioVenn, a visualization tool) showing the number of proteins altered (increased or decreased) by an average of 1.8-fold or

greater in each of the three data sets and the overlap across the three specimens at each concentration of Aquamin®. **Lower panel**: Venn plot showing a distribution of the common proteins (from the top panels) altered (increased or decreased) by each concentration of Aquamin® with an average of 1.8-fold or greater and each individual specimen. These data provide an indication of variability among individual subjects in their response to different concentrations of Aquamin®. A complete list of these common proteins is presented in Supplementary Table 4.

DSG2: Desmoglein-2; CDH17: Cadherin-17; LYPD8: Ly6/PLAUR domain-containing protein 8.
