# Supplementary material for: Ulcerative Colitis-Derived Colonoid Culture: A Multi-Mineral-Approach to Improve Barrier Protein Expression
Source: Front Cell Dev Biol. 2020 Nov 23;8:577221. doi: 10.3389/fcell.2020.577221 (PMC7719760; doi:10.3389/fcell.2020.577221)
Supplement: Supplementary file 7 [file Table_2.PDF]

Supplement Table 2. Antibodies used for quantitative immunohistochemistry (IHC)

Antibody	Vendor	Catalog #	Dilution	Incubation Time	Retrieval Method
Ms CK20 mAb clone K_s20.8	Dako	M7019	1:100	60 min	FLEX TRS High pH 9.0, 20 min
Rb Desmoglein-2 pAb	Sigma	HPA004896	1:200	30 min	[a]HIER pH 6.0
Rb Ki-67 mAb clone SP6	Cell Marque	275R-16	1:250	30 min	[b]HIER pH 9.0, 20 min
Rb LI Cadherin (CDH17) mAb clone EPR3996	abcam	Ab109190	1:250	60 min	[b]HIER pH 9.0

[a]HIER pH 6: Heat induced epitope retrieval Citrate buffer pH6

[b]HIER pH 9: Heat induced epitope retrieval 10 mM Tris HCl/1 mM EDTA buffer pH9
