# Supplementary material for: Ulcerative Colitis-Derived Colonoid Culture: A Multi-Mineral-Approach to Improve Barrier Protein Expression
Source: Front Cell Dev Biol. 2020 Nov 23;8:577221. doi: 10.3389/fcell.2020.577221 (PMC7719760; doi:10.3389/fcell.2020.577221)
Supplement: Supplementary file 8 [file Table_3.PDF]

Supplement Table 3. Statistical Analysis - p Value evaluation by GraphPad Prism 8.3

Figure 1

Lumen size:

ANOVA	p Value	0.0018			
Dunnett's multiple comparisons test	Mean Diff.	95.00% CI of diff.	Significant?	Summary	Adjusted p Value
LWRN25% vs. Aquamin (1.5 mM Ca)	-36.88	-64.19 to -9.576	Yes	**	0.0038
LWRN25% vs. Aquamin (2.1 mM Ca)	-27.9	-54.41 to -1.392	Yes	*	0.0356
LWRN25% vs. Aquamin (3.0 mM Ca)	-43.25	-73.15 to -13.35	Yes	**	0.0017
LWRN25% vs. Aquamin (4.5 mM Ca)	-38.78	-67.18 to -10.38	Yes	**	0.0034

Wall Thickness:

ANOVA	p Value	0.0005			
Dunnett's multiple comparisons test	Mean Diff.	95.00% CI of diff.	Significant?	Summary	Adjusted p Value
LWRN25% vs. Aquamin (1.5 mM Ca)	-3.976	-6.908 to -1.043	Yes	**	0.0037
LWRN25% vs. Aquamin (2.1 mM Ca)	-4.861	-7.708 to -2.014	Yes	***	0.0001
LWRN25% vs. Aquamin (3.0 mM Ca)	-1.864	-5.075 to 1.347	No	ns	0.4135
LWRN25% vs. Aquamin (4.5 mM Ca)	-2.881	-5.931 to 0.1690	No	ns	0.0702

CK20 qIHC:

ANOVA	p Value	<0.0001			
Dunnett's multiple comparisons test	Mean Diff.	95.00% CI of diff.	Significant?	Summary	Adjusted p Value
LWRN25% vs. Aquamin (1.5 mM Ca)	-0.07717	-0.1127 to -0.04165	Yes	****	<0.0001
LWRN25% vs. Aquamin (2.1 mM Ca)	-0.08111	-0.1161 to -0.04616	Yes	****	<0.0001
LWRN25% vs. Aquamin (3.0 mM Ca)	-0.06568	-0.1039 to -0.02748	Yes	***	0.0001
LWRN25% vs. Aquamin (4.5 mM Ca)	-0.08623	-0.1233 to -0.04919	Yes	****	<0.0001

Figure 2

Ki67 qIHC:

ANOVA	p Value	<0.0001			
Dunnett's multiple comparisons test	Mean Diff.	95.00% CI of diff.	Significant?	Summary	Adjusted p Value
LWRN25% vs. Aquamin (1.5 mM Ca)	27.66	19.55 to 35.77	Yes	****	<0.0001
LWRN25% vs. Aquamin (2.1 mM Ca)	22.58	14.68 to 30.49	Yes	****	<0.0001
LWRN25% vs. Aquamin (3.0 mM Ca)	20.93	12.32 to 29.54	Yes	****	<0.0001
LWRN25% vs. Aquamin (4.5 mM Ca)	30.54	21.82 to 39.27	Yes	****	<0.0001

CDH17 qIHC:

ANOVA	p Value	<0.0001			
Dunnett's multiple comparisons test	Mean Diff.	95.00% CI of diff.	Significant?	Summary	Adjusted p Value
LWRN25% vs. Aquamin (1.5 mM Ca)	-0.04806	-0.1035 to 0.007392	No	ns	0.1076
LWRN25% vs. Aquamin (2.1 mM Ca)	-0.0959	-0.1495 to -0.04235	Yes	****	<0.0001
LWRN25% vs. Aquamin (3.0 mM Ca)	-0.1281	-0.1853 to -0.07096	Yes	****	<0.0001
LWRN25% vs. Aquamin (4.5 mM Ca)	-0.1915	-0.2476 to -0.1354	Yes	****	<0.0001

Figure 3

DSG2 qIHC:

ANOVA	p Value	<0.0001			
Dunnett's multiple comparisons test	Mean Diff.	95.00% CI of diff.	Significant?	Summary	Adjusted p Value
LWRN25% vs. Aquamin (1.5 mM Ca)	-0.07441	-0.09704 to -0.05178	Yes	****	<0.0001
LWRN25% vs. Aquamin (2.1 mM Ca)	-0.1	-0.1223 to -0.07776	Yes	****	<0.0001
LWRN25% vs. Aquamin (3.0 mM Ca)	-0.08742	-0.1121 to -0.06273	Yes	****	<0.0001
LWRN25% vs. Aquamin (4.5 mM Ca)	-0.1607	-0.1856 to -0.1357	Yes	****	<0.0001

Desmosome qTEM:

ANOVA	p Value	0.0033			
Dunnett's multiple comparisons test	Mean Diff.	95.00% CI of diff.	Significant?	Summary	Adjusted p Value
LWRN25% vs. Aquamin (1.5 mM Ca)	-1.462	-2.510 to -0.4141	Yes	**	0.0044
LWRN25% vs. Aquamin (3.0 mM Ca)	-1.346	-2.402 to -0.2902	Yes	**	0.0098
