# Supplementary material for: Ulcerative Colitis-Derived Colonoid Culture: A Multi-Mineral-Approach to Improve Barrier Protein Expression
Source: Front Cell Dev Biol. 2020 Nov 23;8:577221. doi: 10.3389/fcell.2020.577221 (PMC7719760; doi:10.3389/fcell.2020.577221)
Supplement: Supplementary file 9 [file Table_4.pdf]

Supplement Table 4. Altered (and common) proteins among different concentrations of Aquamin®

Proteins	Aquamin			
	1.5mM	2.1mM	3.0mM	4.5mM
**Proteins common with all 4 concentrations (Aquamin 1.5, 2.1, 3.0, 4.5 mM) (3 common proteins)**				
Cadherin-17	*2.83±0.22	*2.95±0.60	*3.24±0.67	*3.62±0.70
Desmoglein-2	*2.07±0.17	*2.10±0.25	*2.28±0.37	*2.36±0.40
Ly6/PLAUR domain-containing protein 8	*2.12±0.22	*2.14±0.50	*2.46±0.40	*2.64±0.93
**Proteins common with 3 concentrations (Aquamin 2.1, 3.0, 4.5 mM) (6 common proteins)**				
Calcium-activated chloride channel regulator 4	*2.03±0.28	*2.24±0.51	*2.31±0.27	*3.01±0.74
Natural resistance-associated macrophage protein 2	*1.91±0.42	*2.20±0.43	*2.56±0.36	*2.68±0.54
CEACAM7	*1.84±0.32	*2.00±0.08	*2.23±0.37	*2.44±0.50
Fibrinogen silencer-binding protein	*0.61±0.09	*0.44±0.10	*0.36±0.03	*0.22±0.13
Neurogranin	*0.54±0.05	*0.38±0.16	*0.30±0.13	*0.18±0.09
Stathmin	*0.58±0.07	*0.47±0.02	*0.37±0.01	*0.29±0.12
**Proteins common with 2 concentrations (Aquamin 3.0, 4.5 mM) (21 common proteins)**				
Zinc transporter ZIP4	*2.01±0.55	*2.08±0.46	*2.63±0.77	*2.83±0.96
Apolipoprotein A-I	1.62±0.61	*1.96±0.46	*2.39±0.28	*2.68±0.30
Nuclear receptor subfamily 0 group B member 1	2.59±1.13	5.71±3.91	4.69±2.45	6.68±4.11
Protein RRP5 homolog (NF-kappa-B-binding protein)	*0.72±0.03	*0.54±0.12	*0.44±0.15	*0.41±0.06
DNA replication licensing factor MCM3	*0.70±0.07	*0.57±0.06	*0.44±0.07	*0.34±0.13
Heterogeneous nuclear ribonucleoprotein D-like	*0.69±0.06	*0.59±0.02	*0.50±0.04	*0.42±0.11
Serine/arginine-rich splicing factor 6	*0.67±0.15	*0.56±0.11	*0.47±0.10	*0.37±0.10
Nucleophosmin	*0.59±0.06	*0.55±0.06	*0.43±0.04	*0.35±0.09
Proliferating cell nuclear antigen	*0.65±0.01	*0.59±0.08	*0.45±0.05	*0.36±0.10
Nucleolar RNA helicase 2	*0.63±0.07	*0.52±0.12	*0.43±0.06	*0.34±0.04
Proliferation marker protein Ki-67	*0.58±0.08	*0.48±0.11	*0.39±0.13	*0.31±0.08
Nuclear autoantigenic sperm protein	*0.66±0.07	*0.57±0.03	*0.45±0.03	*0.37±0.11
U3 small nucleolar RNA-interacting protein 2	*0.62±0.03	*0.51±0.06	*0.38±0.01	*0.32±0.10
SWI/SNF complex subunit SMARCC1	*0.69±0.08	*0.54±0.07	*0.48±0.04	*0.41±0.13
Translation machinery-associated protein 7	*0.63±0.07	*0.57±0.10	*0.49±0.06	*0.44±0.09
Nucleolin	*0.70±0.09	*0.57±0.02	*0.48±0.04	*0.41±0.11
Inosine-5'-monophosphate dehydrogenase 2	*0.63±0.03	*0.56±0.13	*0.43±0.11	*0.35±0.12
Nucleolar and coiled-body phosphoprotein 1	*0.63±0.05	*0.53±0.06	*0.44±0.06	*0.37±0.10
PC4 and SFRS1-interacting protein	*0.72±0.10	*0.56±0.05	*0.48±0.05	*0.41±0.12
Receptor-type tyrosine-protein phosphatase F	*0.56±0.09	*0.55±0.11	*0.50±0.08	*0.45±0.07
Cytokine-induced protein with coiled-coil domain	*0.69±0.02	*0.52±0.13	*0.45±0.10	*0.42±0.11
**Proteins unique to 1 concentration (Aquamin 2.1 mM) (7 proteins)**				
Aminopeptidase N	*1.91±0.21	*2.01±0.25	*1.85±0.27	*2.00±0.36
Meprin A subunit alpha	*1.91±0.36	*2.02±0.27	*2.32±0.71	2.37±0.96
CEACAM1	1.72±0.52	5.25±4.20	2.01±1.12	1.80±1.18
CEACAM 5	1.37±0.29	*2.37±0.51	1.81±0.79	1.71±0.82
CEACAM 6	1.25±0.35	*2.13±0.35	1.50±0.78	1.33±0.80
Leucine zipper putative tumor suppressor 1	0.92±0.26	1.54±1.98	1.98±2.19	1.46±0.98
Cyclic AMP-dependent transcription factor ATF-7	*1.37±0.13	*2.25±0.37	*1.76±0.45	1.74±0.57
**Proteins unique to 1 concentration (Aquamin 3.0 mM) (1 protein)**				
Integrin alpha-5	*1.69±0.42	*1.80±0.26	*2.21±0.58	*2.27±0.67
**Proteins unique to 1 concentration (Aquamin 4.5 mM) (33 proteins)**				
Sodium/glucose cotransporter 1	*1.40±0.16	*1.73±0.10	*1.94±0.37	*2.20±0.28
Laminin subunit alpha-1	*2.00±0.62	*2.15±0.38	*2.00±0.49	*2.59±0.44
Laminin subunit beta-1	*2.14±0.70	*2.20±0.48	*2.12±0.56	*2.85±0.42
Laminin subunit beta-2	*1.91±0.49	*2.03±0.34	*1.90±0.30	*2.32±0.30
Laminin subunit gamma-1	*2.03±0.58	*2.07±0.37	*2.01±0.47	*2.68±0.30
Nidogen-1 (Entactin)	1.70±0.49	*1.66±0.37	*1.72±0.37	*2.11±0.28
HMG-CoA synthase, mitochondrial	*1.52±0.12	*1.74±0.23	*1.95±0.22	*2.09±0.18
ENPP3	*1.41±0.15	*1.60±0.18	*1.81±0.19	*2.04±0.25

Copper-transporting ATPase 2	1.39±0.52	*1.62±0.22	*1.94±0.16	*2.28±0.04
Beta-glucuronidase	*1.29±0.06	*1.51±0.23	*1.51±0.17	*2.00±0.27
Keratin, type I cytoskeletal 16	0.93±0.32	0.72±0.37	0.57±0.42	1.37±0.91
Ribosome biogenesis protein BMS1 homolog	0.92±0.32	0.79±0.34	*0.61±0.15	*0.49±0.04
Ras GTPase-activating protein-binding protein 1	*0.75±0.07	*0.61±0.10	*0.55±0.09	*0.47±0.10
HEAT repeat-containing protein 1	*0.69±0.17	*0.64±0.08	*0.55±0.07	*0.43±0.03
DNA replication licensing factor MCM4	*0.70±0.02	*0.67±0.09	*0.52±0.07	*0.43±0.10
Non-histone chromosomal protein HMG-17	*0.73±0.05	*0.55±0.18	*0.49±0.16	*0.43±0.13
Ribosome biogenesis protein WDR12	*0.77±0.11	*0.67±0.10	*0.58±0.04	*0.47±0.05
DNA replication licensing factor MCM5	*0.70±0.10	*0.57±0.18	*0.49±0.16	*0.40±0.13
Probable 28S rRNA (NOP2)	*0.74±0.06	*0.60±0.09	*0.51±0.06	*0.43±0.12
Nucleolar GTP-binding protein 2	*0.59±0.01	*0.64±0.15	*0.55±0.04	*0.30±0.08
Putative RNA-binding protein Luc7-like 1	*0.69±0.07	*0.72±0.11	*0.58±0.15	*0.47±0.07
CD44 antigen	*0.61±0.01	*0.58±0.12	*0.48±0.12	*0.40±0.09
DNA replication licensing factor MCM7	*0.77±0.08	0.74±0.23	*0.56±0.23	*0.41±0.12
Palmitoyl-protein thioesterase 1	*0.66±0.03	*0.63±0.05	*0.60±0.03	*0.49±0.05
CD99 antigen	*0.78±0.03	*0.64±0.10	*0.59±0.10	*0.39±0.04
Pumilio homolog 3	0.78±0.25	*0.70±0.06	*0.46±0.14	*0.33±0.19
Heterogeneous nuclear ribonucleoprotein A0	*0.71±0.09	*0.55±0.19	*0.49±0.20	*0.39±0.16
DNA replication licensing factor MCM2	*0.70±0.06	*0.62±0.16	*0.47±0.15	*0.32±0.11
Importin subunit alpha-1	*0.70±0.08	*0.66±0.11	*0.57±0.05	*0.48±0.04
Ribonucleoside-diphosphate reductase large subunit	*0.68±0.06	*0.56±0.21	*0.46±0.14	*0.35±0.15
Serine/threonine-protein kinase VRK1	*0.70±0.07	*0.61±0.11	*0.52±0.11	*0.41±0.12
Gem-associated protein 5	*0.82±0.11	*0.65±0.13	*0.53±0.16	*0.47±0.11
Transformer-2 protein homolog beta	0.85±0.24	*0.56±0.07	*0.53±0.04	*0.48±0.06

These values represent average fold change (of abundance ratio) compared to the control condition (0.25mM calcium) ± SD. These proteins were up-regulated by 1.8-fold or greater across the three colonoids (subjects). *Represents significance in up- or down-regulation as compared to the control at $p<0.05$ level. These data are also shown in Supplementary Figure 3.
