# Supplementary material for: Ulcerative Colitis-Derived Colonoid Culture: A Multi-Mineral-Approach to Improve Barrier Protein Expression
Source: Front Cell Dev Biol. 2020 Nov 23;8:577221. doi: 10.3389/fcell.2020.577221 (PMC7719760; doi:10.3389/fcell.2020.577221)
Supplement: Supplementary file 10 [file Table_5.pdf]

**Supplement Table 5. Significantly altered proteins with Aquamin® (p value <0.05 across all conditions and subjects)**

| Proteins | Gene | Aquamin 1.5mM | 2.1mM | 3.0mM | 4.5mM |
|---|---|---|---|---|---|
| Cadherin-17 | CDH17 | 2.834±0.216 | 2.952±0.600 | 3.235±0.672 | 3.616±0.703 |
| Zinc transporter ZIP4 | SLC39A4 | 2.012±0.545 | 2.077±0.457 | 2.628±0.769 | 2.833±0.958 |
| Calcium-activated chloride channel regulator 4 | CLCA4 | 2.029±0.281 | 2.236±0.506 | 2.311±0.269 | 3.006±0.742 |
| Natural resistance-associated macrophage protein 2 | SLC11A2 | 1.911±0.419 | 2.203±0.427 | 2.564±0.357 | 2.683±0.543 |
| Desmoglein-2 | DSG2 | 2.069±0.172 | 2.104±0.252 | 2.283±0.367 | 2.356±0.396 |
| Ly6/PLAUR domain-containing protein 8 | LYPD8 | 2.117±0.215 | 2.137±0.502 | 2.459±0.396 | 2.642±0.927 |
| Sterol 26-hydroxylase, mitochondrial | CYP27A1 | 1.791±0.351 | 2.018±0.308 | 2.156±0.416 | 2.233±0.574 |
| Chloride anion exchanger | SLC26A3 | 1.773±0.291 | 1.911±0.426 | 1.889±0.284 | 2.098±0.385 |
| Carcinoembryonic antigen-related cell adhesion molecule 7 | CEACAM7 | 1.835±0.322 | 1.998±0.077 | 2.227±0.372 | 2.438±0.503 |
| Sodium/glucose cotransporter 1 | SLC5A1 | 1.398±0.161 | 1.726±0.098 | 1.940±0.375 | 2.204±0.283 |
| Tissue alpha-L-fucosidase | FUCA1 | 1.617±0.201 | 1.812±0.125 | 1.902±0.270 | 2.065±0.379 |
| Aminopeptidase N | ANPEP | 1.909±0.213 | 2.010±0.247 | 1.854±0.271 | 2.002±0.361 |
| Inter-alpha-trypsin inhibitor heavy chain H4 | ITIH4 | 1.456±0.076 | 1.776±0.310 | 1.791±0.320 | 1.977±0.195 |
| Hydroxymethylglutaryl-CoA synthase, mitochondrial | HMGCS2 | 1.520±0.122 | 1.742±0.225 | 1.954±0.222 | 2.089±0.179 |
| Dehydrogenase/reductase SDR family member 7 | DHRS7 | 1.407±0.176 | 1.569±0.184 | 1.683±0.225 | 1.838±0.316 |
| Ectonucleotide pyrophosphatase/phosphodiesterase family member 3 | ENPP3 | 1.407±0.150 | 1.598±0.182 | 1.812±0.194 | 2.038±0.250 |
| Transmembrane protease serine 2 | TMPRSS2 | 1.532±0.109 | 1.627±0.196 | 1.828±0.180 | 2.077±0.472 |
| Cadherin-related family member 5 | CDHR5 | 1.507±0.143 | 1.613±0.148 | 1.768±0.230 | 1.949±0.478 |
| ATP-binding cassette sub-family G member 2 | ABCG2 | 1.558±0.140 | 1.761±0.089 | 1.781±0.142 | 1.804±0.219 |
| Beta-glucuronidase | GUSB | 1.287±0.058 | 1.513±0.228 | 1.510±0.170 | 2.002±0.270 |
| Glycogen phosphorylase, brain form | PYGB | 1.271±0.081 | 1.412±0.182 | 1.577±0.225 | 1.667±0.270 |
| Fucose mutarotase | FUOM | 1.436±0.133 | 1.759±0.253 | 1.832±0.186 | 2.041±0.575 |
| Solute carrier family 15 member 1 | SLC15A1 | 1.556±0.094 | 1.675±0.138 | 1.725±0.147 | 1.806±0.361 |
| Multidrug resistance protein 1 | ABCB1 | 1.297±0.032 | 1.451±0.039 | 1.547±0.091 | 1.698±0.216 |
| Junction plakoglobin | JUP | 1.597±0.176 | 1.712±0.118 | 1.768±0.130 | 1.774±0.111 |
| P2X purinoceptor 4 | P2RX4 | 1.411±0.135 | 1.516±0.167 | 1.567±0.130 | 1.630±0.267 |
| Long-chain-fatty-acid--CoA ligase 5 | ACSL5 | 1.369±0.155 | 1.451±0.165 | 1.582±0.202 | 1.649±0.297 |
| Hephaestin | HEPH | 1.501±0.173 | 1.647±0.280 | 1.830±0.261 | 1.833±0.264 |
| Amine oxidase [flavin-containing] A | MAOA | 1.344±0.135 | 1.457±0.156 | 1.475±0.174 | 1.580±0.262 |
| Sulfotransferase family cytosolic 1B member 1 | SULT1B1 | 1.533±0.184 | 1.585±0.199 | 1.680±0.124 | 1.736±0.367 |
| Beta-galactosidase | GLB1 | 1.321±0.066 | 1.462±0.087 | 1.581±0.171 | 1.619±0.180 |
| Peroxiredoxin-like 2A | FAM213A | 1.296±0.062 | 1.469±0.142 | 1.527±0.138 | 1.597±0.184 |
| Beta-hexosaminidase subunit beta | HEXB | 1.296±0.027 | 1.365±0.066 | 1.486±0.129 | 1.554±0.169 |

| | | | | | |
|---|---|---|---|---|---|
| Peroxisomal acyl-coenzyme A oxidase 2 | ACOX2 | 1.316±0.037 | 1.418±0.092 | 1.490±0.128 | 1.564±0.188 |
| Guanine nucleotide-binding protein subunit alpha-11 | GNA11 | 1.302±0.067 | 1.439±0.150 | 1.562±0.251 | 1.57±0.210 |
| Superoxide dismutase, mitochondrial | SOD2 | 1.272±0.106 | 1.427±0.141 | 1.418±0.065 | 1.556±0.007 |
| Unconventional myosin-VIIb | MYO7B | 1.259±0.024 | 1.371±0.100 | 1.465±0.133 | 1.476±0.158 |
| Gelsolin | GSN | 1.218±0.065 | 1.231±0.072 | 1.360±0.088 | 1.478±0.175 |
| Ectonucleoside triphosphate diphosphohydrolase 8 | ENTPD8 | 1.489±0.061 | 1.455±0.119 | 1.509±0.076 | 1.608±0.144 |
| Inositol 1,4,5-trisphosphate receptor type 3 | ITPR3 | 1.244±0.055 | 1.359±0.124 | 1.442±0.121 | 1.450±0.130 |
| Diphosphoinositol polyphosphate phosphohydrolase 2 | NUDT4 | 1.216±0.057 | 1.301±0.092 | 1.437±0.043 | 1.587±0.208 |
| Ubiquitin-like protein 3 | UBL3 | 1.320±0.056 | 1.456±0.078 | 1.491±0.076 | 1.502±0.182 |
| Plectin | PLEC | 1.273±0.096 | 1.454±0.077 | 1.434±0.142 | 1.506±0.027 |
| Serotransferrin | TF | 1.375±0.034 | 1.442±0.160 | 1.497±0.219 | 1.568±0.271 |
| Peroxisomal acyl-coenzyme A oxidase 1 | ACOX1 | 1.252±0.093 | 1.243±0.091 | 1.373±0.082 | 1.457±0.081 |
| Lysosomal alpha-glucosidase | GAA | 1.256±0.035 | 1.314±0.036 | 1.460±0.029 | 1.492±0.043 |
| Keratin, type II cytoskeletal 8 | KRT8 | 1.250±0.098 | 1.524±0.092 | 1.433±0.037 | 1.488±0.103 |
| Very-long-chain 3-oxoacyl-CoA reductase | HSD17B12 | 1.284±0.014 | 1.418±0.135 | 1.532±0.110 | 1.533±0.142 |
| Alpha-methylacyl-CoA racemase | AMACR | 1.269±0.050 | 1.439±0.024 | 1.428±0.032 | 1.419±0.076 |
| Ectonucleoside triphosphate diphosphohydrolase 5 | ENTPD5 | 1.257±0.095 | 1.336±0.041 | 1.342±0.092 | 1.387±0.153 |
| Desmocollin-2 | DSC2 | 1.386±0.072 | 1.345±0.109 | 1.385±0.134 | 1.427±0.123 |
| Procollagen-lysine,2-oxoglutarate 5-dioxygenase 2 | PLOD2 | 1.186±0.070 | 1.288±0.125 | 1.406±0.055 | 1.429±0.062 |
| Cytosolic 10-formyltetrahydrofolate dehydrogenase | ALDH1L1 | 1.143±0.054 | 1.210±0.051 | 1.318±0.088 | 1.376±0.127 |
| Pituitary tumor-transforming gene 1 protein | PTTG1IP | 1.325±0.069 | 1.225±0.052 | 1.375±0.022 | 1.399±0.166 |
| Medium-chain specific acyl-CoA dehydrogenase, mitochondrial | ACADM | 1.164±0.058 | 1.309±0.039 | 1.323±0.066 | 1.362±0.136 |
| UDP-glucuronosyltransferase 1-10 | UGT1A10 | 1.298±0.131 | 1.327±0.143 | 1.388±0.033 | 1.394±0.138 |
| Chloride intracellular channel protein 3 | CLIC3 | 1.373±0.143 | 1.328±0.135 | 1.504±0.199 | 1.500±0.240 |
| Major vault protein | MVP | 1.205±0.087 | 1.283±0.093 | 1.319±0.086 | 1.393±0.169 |
| Lysosome membrane protein 2 | SCARB2 | 1.261±0.084 | 1.295±0.123 | 1.366±0.149 | 1.384±0.120 |
| Carnitine O-palmitoyltransferase 1, liver isoform | CPT1A | 1.196±0.078 | 1.278±0.062 | 1.353±0.102 | 1.360±0.145 |
| Calcium-binding mitochondrial carrier protein Aralar2 | SLC25A13 | 1.152±0.058 | 1.288±0.093 | 1.320±0.110 | 1.380±0.133 |
| Acid sphingomyelinase-like phosphodiesterase 3a | SMPDL3A | 1.411±0.102 | 1.356±0.137 | 1.356±0.014 | 1.327±0.130 |
| Acyl-CoA:lysophosphatidylglycerol acyltransferase 1 | LPGAT1 | 1.174±0.045 | 1.263±0.090 | 1.304±0.022 | 1.331±0.105 |
| Xanthine dehydrogenase/oxidase | XDH | 1.267±0.039 | 1.345±0.082 | 1.384±0.052 | 1.403±0.059 |
| Phytanoyl-CoA dioxygenase, peroxisomal | PHYH | 1.119±0.025 | 1.275±0.115 | 1.321±0.068 | 1.357±0.127 |
| Sigma intracellular receptor 2 | TMEM97 | 1.304±0.120 | 1.484±0.123 | 1.396±0.090 | 1.325±0.124 |
| Very long-chain specific acyl-CoA dehydrogenase, mitochondrial | ACADVL | 1.143±0.050 | 1.326±0.046 | 1.363±0.052 | 1.375±0.051 |
| Phosphatidate cytidylyltransferase 1 | CDS1 | 1.126±0.014 | 1.283±0.121 | 1.271±0.090 | 1.309±0.091 |
| Pyruvate carboxylase, mitochondrial | PC | 1.290±0.031 | 1.331±0.155 | 1.360±0.162 | 1.345±0.134 |
| Unconventional myosin-XVB | MYO15B | 1.257±0.093 | 1.246±0.068 | 1.322±0.011 | 1.344±0.133 |
| Protein phosphatase 1 regulatory subunit 3G | PPP1R3G | 1.239±0.035 | 1.265±0.046 | 1.394±0.187 | 1.325±0.114 |

| | | | | | |
|---|---|---|---|---|---|
| DnaJ homolog subfamily A member 4 | DNAJA4 | 1.254±0.107 | 1.212±0.066 | 1.336±0.041 | 1.401±0.138 |
| Procollagen lysine hydroxylase and glycosyltransferase LH3 | PLOD3 | 1.147±0.047 | 1.193±0.047 | 1.294±0.097 | 1.334±0.111 |
| Thiosulfate sulfurtransferase | TST | 1.209±0.013 | 1.273±0.111 | 1.357±0.112 | 1.420±0.203 |
| ATP-dependent 6-phosphofructokinase, liver type | PFKL | 1.143±0.044 | 1.193±0.082 | 1.278±0.110 | 1.276±0.083 |
| Polypeptide N-acetylgalactosaminyltransferase 12 | GALNT12 | 1.196±0.060 | 1.266±0.089 | 1.297±0.089 | 1.287±0.112 |
| Carboxylesterase 3 | CES3 | 1.240±0.101 | 1.313±0.043 | 1.245±0.030 | 1.311±0.120 |
| Serine beta-lactamase-like protein LACTB, mitochondrial | LACTB | 1.176±0.049 | 1.307±0.105 | 1.288±0.034 | 1.313±0.026 |
| Basement membrane-specific heparan sulfate proteoglycan protein | HSPG2 | 1.131±0.013 | 1.227±0.040 | 1.237±0.050 | 1.316±0.104 |
| Maestro heat-like repeat-containing protein family member 1 | MROH1 | 1.135±0.027 | 1.315±0.022 | 1.354±0.060 | 1.331±0.092 |
| ATP-binding cassette sub-family D member 3 | ABCD3 | 1.176±0.046 | 1.306±0.138 | 1.357±0.095 | 1.307±0.076 |
| Mitochondrial proton/calcium exchanger protein | LETM1 | 1.140±0.050 | 1.173±0.069 | 1.211±0.085 | 1.251±0.097 |
| Aldose 1-epimerase | GALM | 1.157±0.052 | 1.169±0.046 | 1.262±0.084 | 1.294±0.049 |
| Aldehyde dehydrogenase, mitochondrial | ALDH2 | 1.167±0.031 | 1.240±0.016 | 1.252±0.037 | 1.282±0.063 |
| IST1 homolog | IST1 | 1.187±0.004 | 1.127±0.030 | 1.243±0.035 | 1.261±0.115 |
| Acyl-coenzyme A thioesterase 1 | ACOT1 | 1.162±0.037 | 1.239±0.022 | 1.248±0.012 | 1.238±0.047 |
| 2,4-dienoyl-CoA reductase, mitochondrial | DECR1 | 1.120±0.029 | 1.127±0.017 | 1.177±0.008 | 1.216±0.079 |
| Serpin H1 | SERPINH1 | 1.132±0.028 | 1.192±0.027 | 1.192±0.061 | 1.228±0.042 |
| Protein mono-ADP-ribosyltransferase PARP4 | PARP4 | 1.173±0.024 | 1.178±0.014 | 1.235±0.047 | 1.273±0.104 |
| Peroxisomal acyl-coenzyme A oxidase 3 | ACOX3 | 1.168±0.049 | 1.193±0.019 | 1.247±0.038 | 1.206±0.024 |
| StAR-related lipid transfer protein 5 | STARD5 | 1.204±0.065 | 1.221±0.091 | 1.297±0.089 | 1.221±0.094 |
| MAGUK p55 subfamily member 7 | MPP7 | 1.052±0.017 | 1.146±0.047 | 1.108±0.018 | 1.183±0.052 |
| Methylglutaconyl-CoA hydratase, mitochondrial | AUH | 1.162±0.038 | 1.246±0.006 | 1.282±0.062 | 1.241±0.092 |
| Farnesyl pyrophosphate synthase | FDPS | 1.137±0.013 | 1.242±0.088 | 1.241±0.068 | 1.229±0.087 |
| Catechol O-methyltransferase domain-containing protein 1 | COMTD1 | 1.208±0.026 | 1.265±0.063 | 1.216±0.036 | 1.215±0.068 |
| Glycogen debranching enzyme | AGL | 1.088±0.026 | 1.121±0.045 | 1.137±0.035 | 1.171±0.033 |
| Succinate dehydrogenase [ubiquinone] flavoprotein, mitochondrial | SDHA | 1.073±0.003 | 1.124±0.051 | 1.171±0.042 | 1.176±0.066 |
| Neuroplastin | NPTN | 1.155±0.013 | 1.140±0.012 | 1.144±0.008 | 1.181±0.065 |
| Vinculin | VCL | 1.095±0.015 | 1.237±0.065 | 1.178±0.060 | 1.188±0.049 |
| Villin-1 | VIL1 | 1.092±0.025 | 1.123±0.035 | 1.142±0.014 | 1.144±0.051 |
| Disintegrin and metalloproteinase domain-containing protein 9 | ADAM9 | 0.903±0.036 | 0.872±0.020 | 0.916±0.007 | 0.899±0.028 |
| Aspartate--tRNA ligase, mitochondrial | DARS2 | 0.949±0.017 | 0.906±0.011 | 0.880±0.018 | 0.875±0.029 |
| E3 ubiquitin-protein ligase HUWE1 | HUWE1 | 0.919±0.030 | 0.900±0.026 | 0.903±0.019 | 0.862±0.044 |
| Integrin alpha-3 | ITGA3 | 0.919±0.027 | 0.903±0.028 | 0.859±0.010 | 0.868±0.035 |
| Talin-1 | TLN1 | 0.919±0.012 | 0.892±0.016 | 0.881±0.020 | 0.867±0.050 |
| Nuclear pore complex protein Nup155 | NUP155 | 0.832±0.011 | 0.832±0.059 | 0.800±0.063 | 0.801±0.046 |
| Omega-amidase NIT2 | NIT2 | 0.859±0.027 | 0.828±0.055 | 0.844±0.016 | 0.821±0.017 |
| Echinoderm microtubule-associated protein-like 4 | EML4 | 0.872±0.011 | 0.879±0.017 | 0.848±0.022 | 0.819±0.029 |
| Hsp90 co-chaperone Cdc37 | CDC37 | 0.907±0.030 | 0.864±0.046 | 0.839±0.025 | 0.831±0.029 |

| | | | | | |
|---|---|---|---|---|---|
| Eukaryotic translation initiation factor 2 subunit 1 | EIF2S1 | 0.879±0.039 | 0.853±0.034 | 0.835±0.039 | 0.788±0.050 |
| Peptidyl-prolyl cis-trans isomerase D | PPID | 0.891±0.019 | 0.848±0.024 | 0.800±0.028 | 0.788±0.070 |
| Constitutive coactivator of PPAR-gamma-like protein 1 | FAM120A | 0.933±0.021 | 0.901±0.013 | 0.893±0.040 | 0.816±0.024 |
| Elongation factor 2 | EEF2 | 0.880±0.005 | 0.847±0.051 | 0.803±0.035 | 0.787±0.057 |
| RNA-binding protein Musashi homolog 2 | MSI2 | 0.913±0.022 | 0.829±0.043 | 0.800±0.043 | 0.807±0.027 |
| Kynurenine--oxoglutarate transaminase 3 | KYAT3 | 0.865±0.038 | 0.849±0.019 | 0.845±0.011 | 0.790±0.044 |
| U6 snRNA-associated Sm-like protein LSm6 | LSM6 | 0.843±0.053 | 0.858±0.016 | 0.800±0.039 | 0.755±0.071 |
| Elongation factor 1-alpha 1 | EEF1A1 | 0.871±0.010 | 0.858±0.046 | 0.780±0.052 | 0.750±0.076 |
| Cell cycle and apoptosis regulator protein 2 | CCAR2 | 0.855±0.036 | 0.846±0.046 | 0.788±0.047 | 0.760±0.053 |
| Stress-induced-phosphoprotein 1 | STIP1 | 0.878±0.024 | 0.791±0.048 | 0.780±0.060 | 0.773±0.071 |
| Eukaryotic translation initiation factor 3 subunit K | EIF3K | 0.903±0.029 | 0.810±0.022 | 0.817±0.045 | 0.786±0.027 |
| Regulator of nonsense transcripts 1 | UPF1 | 0.904±0.020 | 0.854±0.017 | 0.832±0.017 | 0.788±0.043 |
| 2'-deoxynucleoside 5'-phosphate N-hydrolase 1 | DNPH1 | 0.873±0.013 | 0.834±0.035 | 0.802±0.036 | 0.768±0.054 |
| Eukaryotic translation initiation factor 6 | EIF6 | 0.868±0.030 | 0.815±0.037 | 0.804±0.021 | 0.770±0.020 |
| Tubulin beta-4B chain | TUBB4B | 0.872±0.022 | 0.882±0.036 | 0.832±0.031 | 0.785±0.031 |
| Thioredoxin domain-containing protein 5 | TXNDC5 | 0.882±0.014 | 0.814±0.028 | 0.803±0.030 | 0.789±0.071 |
| Eukaryotic translation initiation factor 3 subunit A | EIF3A | 0.849±0.025 | 0.831±0.044 | 0.786±0.036 | 0.727±0.068 |
| Glycylpeptide N-tetradecanoyltransferase 1 | NMT1 | 0.869±0.033 | 0.842±0.005 | 0.843±0.003 | 0.751±0.032 |
| Actin-like protein 6A | ACTL6A | 0.890±0.013 | 0.863±0.042 | 0.794±0.029 | 0.779±0.054 |
| Paraspeckle component 1 | PSPC1 | 0.853±0.048 | 0.811±0.063 | 0.785±0.058 | 0.720±0.088 |
| Peptidyl-prolyl cis-trans isomerase FKBP4 | FKBP4 | 0.860±0.036 | 0.827±0.054 | 0.766±0.067 | 0.747±0.067 |
| Splicing factor 3B subunit 3 | SF3B3 | 0.833±0.049 | 0.806±0.061 | 0.752±0.065 | 0.696±0.098 |
| S-adenosylmethionine synthase isoform type-2 | MAT2A | 0.877±0.016 | 0.886±0.033 | 0.783±0.037 | 0.759±0.037 |
| Signal recognition particle subunit SRP72 | SRP72 | 0.898±0.011 | 0.858±0.051 | 0.840±0.037 | 0.753±0.022 |
| Cold shock domain-containing protein E1 | CSDE1 | 0.901±0.024 | 0.866±0.037 | 0.795±0.005 | 0.754±0.057 |
| Polyadenylate-binding protein 1 | PABPC1 | 0.826±0.012 | 0.788±0.071 | 0.736±0.039 | 0.713±0.071 |
| High mobility group protein HMG-I/HMG-Y | HMGA1 | 0.841±0.024 | 0.784±0.076 | 0.772±0.066 | 0.753±0.070 |
| Pre-mRNA-processing factor 40 homolog A | PRPF40A | 0.848±0.052 | 0.787±0.033 | 0.804±0.056 | 0.767±0.062 |
| Secretory carrier-associated membrane protein 3 | SCAMP3 | 0.894±0.022 | 0.873±0.033 | 0.810±0.061 | 0.762±0.063 |
| Protein FAM50A | FAM50A | 0.820±0.051 | 0.814±0.046 | 0.764±0.045 | 0.736±0.052 |
| NADH dehydrogenase [ubiquinone] 1 alpha assembly factor 2 | NDUFAF2 | 0.795±0.016 | 0.792±0.034 | 0.731±0.018 | 0.700±0.080 |
| Chromatin complexes subunit BAP18 | BAP18 | 0.856±0.044 | 0.752±0.069 | 0.723±0.066 | 0.708±0.076 |
| Actin-histidine N-methyltransferase | SETD3 | 0.843±0.051 | 0.833±0.030 | 0.737±0.051 | 0.706±0.058 |
| Syndecan-1 | SDC1 | 0.771±0.040 | 0.736±0.056 | 0.731±0.048 | 0.755±0.061 |
| Branched-chain-amino-acid aminotransferase, mitochondrial | BCAT2 | 0.814±0.034 | 0.824±0.024 | 0.791±0.048 | 0.735±0.068 |
| KH domain-containing, RNA-binding, signal transduction protein 1 | KHDRBS1 | 0.808±0.039 | 0.782±0.003 | 0.753±0.005 | 0.712±0.056 |
| Acidic leucine-rich nuclear phosphoprotein 32 family member A | ANP32A | 0.844±0.052 | 0.727±0.034 | 0.733±0.029 | 0.718±0.031 |
| Prefoldin subunit 5 | PFDN5 | 0.838±0.016 | 0.705±0.096 | 0.723±0.092 | 0.681±0.091 |

| | | | | | |
|---|---|---|---|---|---|
| Translation initiation factor eIF-2B subunit gamma | EIF2B3 | 0.823±0.044 | 0.721±0.081 | 0.710±0.047 | 0.680±0.072 |
| U4/U6.U5 tri-snRNP-associated protein 1 | SART1 | 0.848±0.037 | 0.792±0.026 | 0.755±0.020 | 0.709±0.059 |
| Eukaryotic translation initiation factor 3 subunit J | EIF3J | 0.842±0.027 | 0.768±0.029 | 0.728±0.030 | 0.678±0.077 |
| Heterogeneous nuclear ribonucleoprotein U-like protein 1 | HNRNPUL1 | 0.832±0.054 | 0.802±0.034 | 0.770±0.044 | 0.711±0.087 |
| Zinc finger RNA-binding protein | ZFR | 0.852±0.012 | 0.765±0.075 | 0.751±0.074 | 0.716±0.097 |
| Leucine-rich repeat-containing protein 40 | LRRC40 | 0.855±0.054 | 0.803±0.027 | 0.776±0.006 | 0.708±0.008 |
| WD40 repeat-containing protein SMU1 | SMU1 | 0.833±0.047 | 0.883±0.017 | 0.755±0.054 | 0.692±0.099 |
| Mitochondrial-processing peptidase subunit alpha | PMPCA | 0.803±0.068 | 0.819±0.032 | 0.776±0.030 | 0.718±0.058 |
| RNA transcription, translation and transport factor protein | RTRAF | 0.861±0.040 | 0.788±0.035 | 0.764±0.037 | 0.728±0.055 |
| Serrate RNA effector molecule homolog | SRRT | 0.814±0.063 | 0.776±0.064 | 0.744±0.064 | 0.692±0.091 |
| Translation initiation factor eIF-2B subunit epsilon | EIF2B5 | 0.842±0.053 | 0.820±0.032 | 0.745±0.071 | 0.686±0.087 |
| Rab GTPase-binding effector protein 1 | RABEP1 | 0.825±0.037 | 0.817±0.024 | 0.741±0.007 | 0.710±0.021 |
| E3 SUMO-protein ligase RanBP2 | RANBP2 | 0.861±0.011 | 0.830±0.033 | 0.832±0.055 | 0.730±0.074 |
| RNA-binding protein EWS | EWSR1 | 0.857±0.051 | 0.767±0.040 | 0.735±0.009 | 0.688±0.045 |
| Heterogeneous nuclear ribonucleoprotein H | HNRNPH1 | 0.848±0.032 | 0.760±0.051 | 0.733±0.046 | 0.686±0.078 |
| Peptidyl-prolyl cis-trans isomerase FKBP3 | FKBP3 | 0.804±0.053 | 0.751±0.073 | 0.713±0.068 | 0.687±0.099 |
| Eukaryotic translation initiation factor 4E type 2 | EIF4E2 | 0.823±0.032 | 0.805±0.063 | 0.752±0.021 | 0.683±0.081 |
| Splicing factor, proline- and glutamine-rich | SFPQ | 0.833±0.057 | 0.808±0.053 | 0.746±0.052 | 0.677±0.076 |
| Splicing factor 3A subunit 3 | SF3A3 | 0.818±0.041 | 0.777±0.055 | 0.749±0.042 | 0.690±0.077 |
| U2 small nuclear ribonucleoprotein A' | SNRPA1 | 0.808±0.057 | 0.758±0.075 | 0.750±0.075 | 0.654±0.072 |
| 40S ribosomal protein S6 | RPS6 | 0.738±0.058 | 0.743±0.009 | 0.665±0.021 | 0.640±0.085 |
| Alpha-taxilin | TXLNA | 0.842±0.033 | 0.786±0.012 | 0.719±0.085 | 0.682±0.060 |
| 60S ribosomal protein L12 | RPL12 | 0.783±0.027 | 0.769±0.049 | 0.690±0.056 | 0.650±0.082 |
| GMP synthase [glutamine-hydrolyzing] | GMPS | 0.776±0.035 | 0.785±0.060 | 0.702±0.036 | 0.671±0.024 |
| Rap1 GTPase-GDP dissociation stimulator 1 | RAP1GDS1 | 0.810±0.037 | 0.795±0.036 | 0.725±0.015 | 0.677±0.023 |
| U2 small nuclear ribonucleoprotein B'' | SNRPB2 | 0.813±0.012 | 0.760±0.017 | 0.719±0.025 | 0.679±0.028 |
| 40S ribosomal protein S11 | RPS11 | 0.759±0.079 | 0.783±0.061 | 0.692±0.052 | 0.645±0.108 |
| Splicing factor 3A subunit 1 | SF3A1 | 0.827±0.021 | 0.774±0.034 | 0.734±0.025 | 0.684±0.025 |
| E3 ubiquitin-protein ligase RBBP6 | RBBP6 | 0.799±0.007 | 0.691±0.063 | 0.687±0.093 | 0.640±0.085 |
| Heat shock 70 kDa protein 4L | HSPA4L | 0.787±0.050 | 0.738±0.031 | 0.682±0.055 | 0.624±0.109 |
| Splicing factor 3B subunit 5 | SF3B5 | 0.822±0.041 | 0.752±0.070 | 0.686±0.084 | 0.695±0.066 |
| H/ACA ribonucleoprotein complex subunit DKC1 | DKC1 | 0.762±0.031 | 0.742±0.036 | 0.675±0.075 | 0.613±0.096 |
| Band 4.1-like protein 2 | EPB41L2 | 0.807±0.018 | 0.760±0.007 | 0.723±0.011 | 0.681±0.064 |
| SUMO-conjugating enzyme UBC9 | UBE2I | 0.797±0.066 | 0.742±0.057 | 0.712±0.060 | 0.687±0.092 |
| Poly(U)-binding-splicing factor PUF60 | PUF60 | 0.835±0.001 | 0.794±0.062 | 0.714±0.043 | 0.670±0.037 |
| 40S ribosomal protein S17 | RPS17 | 0.798±0.039 | 0.747±0.025 | 0.689±0.011 | 0.648±0.045 |
| Pre-mRNA-processing factor 19 | PRPF19 | 0.795±0.048 | 0.740±0.032 | 0.688±0.033 | 0.635±0.099 |
| tRNA (cytosine(34)-C(5))-methyltransferase | NSUN2 | 0.811±0.031 | 0.793±0.040 | 0.730±0.018 | 0.661±0.050 |

| | | | | | |
|---|---|---|---|---|---|
| General transcription factor IIF subunit 1 | GTF2F1 | 0.804±0.039 | 0.766±0.034 | 0.687±0.017 | 0.677±0.067 |
| C-1-tetrahydrofolate synthase, cytoplasmic | MTHFD1 | 0.806±0.048 | 0.752±0.053 | 0.704±0.048 | 0.621±0.065 |
| Caprin-1 | CAPRIN1 | 0.799±0.033 | 0.726±0.042 | 0.676±0.053 | 0.653±0.053 |
| Peptidyl-prolyl cis-trans isomerase G | PPIG | 0.800±0.062 | 0.775±0.078 | 0.780±0.060 | 0.684±0.064 |
| Serine-threonine kinase receptor-associated protein | STRAP | 0.822±0.050 | 0.772±0.013 | 0.721±0.036 | 0.686±0.078 |
| 4F2 cell-surface antigen heavy chain | SLC3A2 | 0.813±0.048 | 0.783±0.034 | 0.686±0.060 | 0.623±0.076 |
| Enhancer of mRNA-decapping protein 3 | EDC3 | 0.729±0.085 | 0.719±0.048 | 0.672±0.061 | 0.666±0.042 |
| Eukaryotic peptide chain release factor GTP-binding subunit ERF3A | GSPT1 | 0.835±0.036 | 0.773±0.005 | 0.706±0.036 | 0.656±0.072 |
| 40S ribosomal protein S5 | RPS5 | 0.841±0.039 | 0.750±0.041 | 0.709±0.081 | 0.685±0.109 |
| Nascent polypeptide-associated complex subunit alpha | NACA | 0.792±0.051 | 0.736±0.073 | 0.644±0.066 | 0.601±0.099 |
| Nucleolar protein 56 | NOP56 | 0.756±0.002 | 0.749±0.069 | 0.682±0.086 | 0.598±0.101 |
| Thymidylate kinase | DTYMK | 0.832±0.007 | 0.774±0.010 | 0.732±0.029 | 0.647±0.050 |
| Zinc finger CCCH domain-containing protein 15 | ZC3H15 | 0.778±0.010 | 0.710±0.018 | 0.676±0.008 | 0.629±0.050 |
| Nascent polypeptide-associated complex subunit alpha-2 | NACA2 | 0.788±0.044 | 0.728±0.053 | 0.640±0.044 | 0.611±0.054 |
| Histone H1.2 | HIST1H1C | 0.722±0.087 | 0.662±0.074 | 0.661±0.067 | 0.591±0.105 |
| Splicing factor 3B subunit 1 | SF3B1 | 0.830±0.050 | 0.796±0.025 | 0.734±0.037 | 0.654±0.108 |
| DnaJ homolog subfamily C member 7 | DNAJC7 | 0.799±0.049 | 0.787±0.037 | 0.692±0.055 | 0.649±0.097 |
| La-related protein 4B | LARP4B | 0.781±0.050 | 0.700±0.020 | 0.674±0.071 | 0.639±0.056 |
| 60S ribosomal protein L18 | RPL18 | 0.760±0.041 | 0.784±0.046 | 0.716±0.046 | 0.629±0.051 |
| Host cell factor 1 | HCFC1 | 0.769±0.071 | 0.766±0.067 | 0.696±0.057 | 0.662±0.105 |
| 60S ribosomal protein L19 | RPL19 | 0.774±0.045 | 0.723±0.045 | 0.684±0.045 | 0.597±0.064 |
| Nuclear pore complex protein Nup98-Nup96 | NUP98 | 0.796±0.035 | 0.705±0.090 | 0.639±0.079 | 0.598±0.046 |
| Clustered mitochondria protein homolog | CLUH | 0.828±0.010 | 0.750±0.047 | 0.668±0.021 | 0.610±0.061 |
| ADP-ribosylation factor-like protein 3 | ARL3 | 0.720±0.057 | 0.781±0.067 | 0.732±0.033 | 0.621±0.024 |
| RNA-binding protein 4 | RBM4 | 0.824±0.027 | 0.729±0.047 | 0.723±0.035 | 0.640±0.099 |
| Bcl-2-associated transcription factor 1 | BCLAF1 | 0.792±0.036 | 0.716±0.016 | 0.668±0.017 | 0.610±0.033 |
| U6 snRNA-associated Sm-like protein LSm2 | LSM2 | 0.778±0.025 | 0.771±0.071 | 0.658±0.016 | 0.617±0.097 |
| Ran GTPase-activating protein 1 | RANGAP1 | 0.772±0.042 | 0.737±0.045 | 0.679±0.016 | 0.630±0.066 |
| U1 small nuclear ribonucleoprotein A | SNRPA | 0.794±0.072 | 0.767±0.027 | 0.703±0.038 | 0.654±0.092 |
| Cyclin-dependent kinase 12 | CDK12 | 0.787±0.068 | 0.727±0.042 | 0.732±0.055 | 0.618±0.068 |
| m7GpppX diphosphatase | DCPS | 0.778±0.032 | 0.733±0.049 | 0.679±0.013 | 0.597±0.061 |
| Eukaryotic translation initiation factor 5A-1 | EIF5A | 0.798±0.034 | 0.730±0.014 | 0.669±0.042 | 0.626±0.087 |
| Serine/arginine repetitive matrix protein 2 | SRRM2 | 0.760±0.031 | 0.703±0.088 | 0.647±0.074 | 0.629±0.074 |
| Matrin-3 | MATR3 | 0.794±0.020 | 0.738±0.048 | 0.684±0.024 | 0.609±0.017 |
| SH3 domain-binding glutamic acid-rich-like protein | SH3BGRL | 0.701±0.049 | 0.611±0.115 | 0.573±0.096 | 0.557±0.108 |
| Poly(rC)-binding protein 2 | PCBP2 | 0.781±0.018 | 0.690±0.063 | 0.662±0.019 | 0.621±0.048 |
| RNA-binding protein 39 | RBM39 | 0.796±0.055 | 0.728±0.063 | 0.669±0.064 | 0.615±0.037 |
| Splicing factor 3B subunit 2 | SF3B2 | 0.819±0.048 | 0.732±0.005 | 0.707±0.005 | 0.628±0.065 |

| | | | | | |
|---|---|---|---|---|---|
| SUMO-activating enzyme subunit 1 | SAE1 | 0.785±0.037 | 0.736±0.027 | 0.698±0.014 | 0.615±0.082 |
| Proline-, glutamic acid- and leucine-rich protein 1 | PELP1 | 0.818±0.033 | 0.767±0.065 | 0.738±0.069 | 0.650±0.106 |
| Heterogeneous nuclear ribonucleoprotein A3 | HNRNPA3 | 0.831±0.046 | 0.720±0.012 | 0.706±0.033 | 0.635±0.080 |
| Protein RCC2 | RCC2 | 0.735±0.047 | 0.745±0.079 | 0.624±0.097 | 0.544±0.124 |
| RNA-binding protein 14 | RBM14 | 0.795±0.018 | 0.688±0.050 | 0.653±0.062 | 0.574±0.072 |
| ELAV-like protein 1 | ELAVL1 | 0.800±0.016 | 0.748±0.026 | 0.678±0.003 | 0.615±0.067 |
| Heterogeneous nuclear ribonucleoprotein K | HNRNPK | 0.800±0.054 | 0.700±0.045 | 0.656±0.055 | 0.596±0.055 |
| 40S ribosomal protein SA | RPSA | 0.793±0.061 | 0.722±0.036 | 0.648±0.043 | 0.615±0.089 |
| La-related protein 1 | LARP1 | 0.801±0.036 | 0.697±0.079 | 0.643±0.071 | 0.599±0.069 |
| Cell division cycle and apoptosis regulator protein 1 | CCAR1 | 0.801±0.036 | 0.704±0.065 | 0.663±0.037 | 0.606±0.065 |
| Nuclear migration protein nudC | NUDC | 0.747±0.026 | 0.692±0.051 | 0.602±0.027 | 0.558±0.065 |
| Tubulin beta chain | TUBB | 0.758±0.031 | 0.715±0.046 | 0.644±0.051 | 0.587±0.024 |
| Heterogeneous nuclear ribonucleoprotein A1 | HNRNPA1 | 0.742±0.039 | 0.664±0.106 | 0.587±0.089 | 0.540±0.103 |
| Lupus La protein | SSB | 0.753±0.060 | 0.669±0.038 | 0.608±0.026 | 0.552±0.085 |
| 40S ribosomal protein S10 | RPS10 | 0.772±0.066 | 0.709±0.048 | 0.638±0.030 | 0.582±0.056 |
| Monofunctional C1-tetrahydrofolate synthase, mitochondrial | MTHFD1L | 0.839±0.037 | 0.791±0.058 | 0.730±0.049 | 0.594±0.093 |
| Proteasome activator complex subunit 3 | PSME3 | 0.786±0.020 | 0.701±0.025 | 0.618±0.023 | 0.563±0.088 |
| DNA mismatch repair protein Msh2 | MSH2 | 0.742±0.085 | 0.709±0.092 | 0.615±0.073 | 0.550±0.112 |
| 28 kDa heat- and acid-stable phosphoprotein | PDAP1 | 0.774±0.065 | 0.685±0.035 | 0.644±0.061 | 0.577±0.089 |
| 40S ribosomal protein S19 | RPS19 | 0.770±0.066 | 0.721±0.064 | 0.647±0.077 | 0.617±0.095 |
| 40S ribosomal protein S12 | RPS12 | 0.748±0.054 | 0.621±0.091 | 0.578±0.104 | 0.574±0.118 |
| Transcription factor BTF3 | BTF3 | 0.773±0.065 | 0.684±0.015 | 0.630±0.061 | 0.566±0.071 |
| Heterogeneous nuclear ribonucleoprotein U | HNRNPU | 0.744±0.069 | 0.643±0.087 | 0.618±0.090 | 0.547±0.121 |
| Solute carrier family 12 member 2 | SLC12A2 | 0.766±0.078 | 0.711±0.020 | 0.661±0.003 | 0.573±0.066 |
| Nuclease-sensitive element-binding protein 1 | YBX1 | 0.783±0.057 | 0.713±0.035 | 0.623±0.049 | 0.555±0.074 |
| Thyroid hormone receptor-associated protein 3 | THRAP3 | 0.764±0.023 | 0.695±0.017 | 0.662±0.009 | 0.588±0.055 |
| DnaJ homolog subfamily C member 8 | DNAJC8 | 0.792±0.069 | 0.706±0.015 | 0.638±0.027 | 0.594±0.107 |
| Scaffold attachment factor B1 | SAFB | 0.781±0.044 | 0.690±0.028 | 0.649±0.032 | 0.589±0.079 |
| Histone-binding protein RBBP4 | RBBP4 | 0.758±0.076 | 0.625±0.034 | 0.621±0.050 | 0.568±0.103 |
| Heterogeneous nuclear ribonucleoprotein M | HNRNPM | 0.797±0.047 | 0.718±0.017 | 0.680±0.022 | 0.588±0.079 |
| General transcription factor II-I | GTF2I | 0.743±0.068 | 0.646±0.024 | 0.587±0.019 | 0.538±0.102 |
| Putative RNA-binding protein Luc7-like 2 | LUC7L2 | 0.716±0.041 | 0.630±0.014 | 0.587±0.020 | 0.538±0.072 |
| Heterogeneous nuclear ribonucleoprotein F | HNRNPF | 0.790±0.045 | 0.692±0.046 | 0.663±0.060 | 0.597±0.091 |
| 60S ribosomal protein L36 | RPL36 | 0.728±0.058 | 0.727±0.018 | 0.660±0.074 | 0.588±0.087 |
| Hydroxymethylglutaryl-CoA synthase, cytoplasmic | HMGCS1 | 0.838±0.059 | 0.778±0.010 | 0.640±0.051 | 0.541±0.120 |
| Apoptosis-associated speck-like protein containing a CARD | PYCARD | 0.786±0.071 | 0.705±0.084 | 0.630±0.057 | 0.594±0.129 |
| Histone H1.5 | HIST1H1B | 0.732±0.071 | 0.663±0.059 | 0.639±0.081 | 0.577±0.104 |
| PEST proteolytic signal-containing nuclear protein | PCNP | 0.714±0.028 | 0.617±0.095 | 0.556±0.084 | 0.544±0.116 |

| | | | | | |
|---|---|---|---|---|---|
| Histone-binding protein RBBP7 | RBBP7 | 0.742±0.051 | 0.675±0.059 | 0.591±0.032 | 0.524±0.075 |
| Plasminogen activator inhibitor 1 RNA-binding protein | SERBP1 | 0.740±0.055 | 0.685±0.042 | 0.599±0.036 | 0.554±0.079 |
| DNA topoisomerase 2-beta | TOP2B | 0.769±0.016 | 0.740±0.062 | 0.676±0.018 | 0.566±0.081 |
| Signal recognition particle 9 kDa protein | SRP9 | 0.740±0.041 | 0.650±0.037 | 0.596±0.012 | 0.528±0.077 |
| Ubiquitin-associated protein 2-like | UBAP2L | 0.720±0.051 | 0.615±0.071 | 0.574±0.043 | 0.550±0.066 |
| Non-POU domain-containing octamer-binding protein | NONO | 0.743±0.081 | 0.699±0.051 | 0.668±0.086 | 0.586±0.131 |
| Treacle protein | TCOF1 | 0.726±0.055 | 0.635±0.047 | 0.587±0.078 | 0.530±0.132 |
| Luc7-like protein 3 | LUC7L3 | 0.768±0.044 | 0.652±0.077 | 0.618±0.044 | 0.531±0.023 |
| Transcription intermediary factor 1-beta | TRIM28 | 0.744±0.037 | 0.625±0.089 | 0.579±0.085 | 0.504±0.107 |
| Chromobox protein homolog 3 | CBX3 | 0.730±0.027 | 0.681±0.017 | 0.600±0.019 | 0.558±0.093 |
| Drebrin | DBN1 | 0.704±0.079 | 0.599±0.045 | 0.585±0.020 | 0.534±0.085 |
| Heterogeneous nuclear ribonucleoprotein D0 | HNRNPD | 0.729±0.069 | 0.641±0.047 | 0.596±0.038 | 0.508±0.089 |
| Ubiquitin-conjugating enzyme E2 E3 | UBE2E3 | 0.725±0.096 | 0.584±0.014 | 0.539±0.046 | 0.524±0.054 |
| RNA-binding motif protein, X chromosome | RBMX | 0.791±0.037 | 0.679±0.056 | 0.619±0.025 | 0.525±0.047 |
| Ras GTPase-activating protein-binding protein 1 | G3BP1 | 0.749±0.074 | 0.611±0.101 | 0.554±0.085 | 0.471±0.098 |
| Palmitoyl-protein thioesterase 1 | PPT1 | 0.658±0.028 | 0.634±0.049 | 0.604±0.030 | 0.489±0.046 |
| DNA repair protein XRCC1 | XRCC1 | 0.730±0.079 | 0.691±0.056 | 0.618±0.112 | 0.564±0.135 |
| Endothelial differentiation-related factor 1 | EDF1 | 0.762±0.075 | 0.617±0.025 | 0.562±0.032 | 0.512±0.078 |
| BRCA2 and CDKN1A-interacting protein | BCCIP | 0.697±0.055 | 0.682±0.074 | 0.566±0.029 | 0.515±0.063 |
| NHP2-like protein 1 | SNU13 | 0.688±0.042 | 0.570±0.076 | 0.529±0.084 | 0.473±0.107 |
| Leydig cell tumor 10 kDa protein homolog | C19orf53 | 0.669±0.010 | 0.633±0.016 | 0.556±0.023 | 0.494±0.069 |
| Multifunctional methyltransferase subunit TRM112-like protein | TRMT112 | 0.725±0.079 | 0.617±0.053 | 0.566±0.059 | 0.478±0.104 |
| Receptor-type tyrosine-protein phosphatase F | PTPRF | 0.559±0.086 | 0.545±0.111 | 0.499±0.075 | 0.449±0.069 |
| Serine/threonine-protein kinase VRK1 | VRK1 | 0.700±0.071 | 0.610±0.109 | 0.524±0.105 | 0.405±0.117 |
| Nucleolin | NCL | 0.697±0.092 | 0.573±0.024 | 0.478±0.043 | 0.409±0.112 |
| Heterogeneous nuclear ribonucleoprotein D-like | HNRNPDL | 0.692±0.064 | 0.585±0.016 | 0.496±0.036 | 0.418±0.107 |
| TATA-binding protein-associated factor 2N | TAF15 | 0.732±0.050 | 0.633±0.012 | 0.569±0.048 | 0.495±0.104 |
| Ribosomal L1 domain-containing protein 1 | RSL1D1 | 0.658±0.003 | 0.592±0.068 | 0.504±0.078 | 0.444±0.136 |
| Probable 28S rRNA (cytosine(4447)-C(5))-methyltransferase | NOP2 | 0.735±0.061 | 0.598±0.085 | 0.513±0.058 | 0.425±0.115 |
| Translation machinery-associated protein 7 | TMA7 | 0.631±0.067 | 0.569±0.098 | 0.485±0.055 | 0.439±0.094 |
| Replication factor C subunit 4 | RFC4 | 0.681±0.106 | 0.635±0.059 | 0.559±0.076 | 0.492±0.114 |
| Deoxyuridine 5'-triphosphate nucleotidohydrolase, mitochondrial | DUT | 0.762±0.058 | 0.600±0.097 | 0.537±0.081 | 0.458±0.146 |
| Proliferating cell nuclear antigen | PCNA | 0.648±0.013 | 0.589±0.079 | 0.451±0.047 | 0.364±0.102 |
| DNA replication licensing factor MCM4 | MCM4 | 0.701±0.018 | 0.666±0.091 | 0.522±0.065 | 0.432±0.097 |
| Nucleolar and coiled-body phosphoprotein 1 | NOLC1 | 0.627±0.051 | 0.534±0.056 | 0.440±0.063 | 0.366±0.104 |
| DNA replication licensing factor MCM3 | MCM3 | 0.697±0.071 | 0.570±0.055 | 0.443±0.068 | 0.343±0.125 |
| Inosine-5'-monophosphate dehydrogenase 2 | IMPDH2 | 0.628±0.029 | 0.559±0.128 | 0.426±0.107 | 0.349±0.115 |
| CD99 antigen | CD99 | 0.781±0.027 | 0.636±0.099 | 0.592±0.096 | 0.386±0.035 |

| | | | | | |
|---|---|---|---|---|---|
| SWI/SNF complex subunit SMARCC1 | SMARCC1 | 0.692±0.075 | 0.543±0.073 | 0.478±0.044 | 0.406±0.126 |
| Nuclear autoantigenic sperm protein | NASP | 0.657±0.069 | 0.565±0.028 | 0.452±0.025 | 0.374±0.110 |
| Coiled-coil domain-containing protein 86 | CCDC86 | 0.688±0.021 | 0.516±0.127 | 0.447±0.102 | 0.418±0.114 |
| CD44 antigen | CD44 | 0.610±0.012 | 0.575±0.121 | 0.482±0.116 | 0.396±0.092 |
| Nucleophosmin | NPM1 | 0.593±0.061 | 0.550±0.063 | 0.427±0.042 | 0.353±0.091 |
| U3 small nucleolar RNA-interacting protein 2 | RRP9 | 0.622±0.026 | 0.505±0.061 | 0.378±0.007 | 0.319±0.098 |
| Nucleolar RNA helicase 2 | DDX21 | 0.632±0.071 | 0.519±0.118 | 0.425±0.064 | 0.341±0.041 |
| Stathmin | STMN1 | 0.576±0.072 | 0.466±0.019 | 0.371±0.006 | 0.285±0.122 |
| Proliferation marker protein Ki-67 | MKI67 | 0.576±0.084 | 0.482±0.113 | 0.391±0.130 | 0.314±0.083 |

These values represent average fold change (of abundance ratio) compared with control condition (0.25mM calcium) ±SD. These proteins were up- or down-regulated and were significant ($p$<0.05) across the three colonoids (subjects) and all culture conditions based on $p$ value (<0.05) calculated by nested-designed experiments and as compared to control. False Discovery rate (FDR) was <1% for all the proteins presented here. These data are also shown in a heatmap - Figure 4A.
